# Amelioration of amyloid-β-induced deficits by DcR3 in an Alzheimer’s disease model

**DOI:** 10.1186/s13024-017-0173-0

**Published:** 2017-04-24

**Authors:** Yi-Ling Liu, Wei-Ting Chen, Yu-Yi Lin, Po-Hung Lu, Shie-Liang Hsieh, Irene Han-Juo Cheng

**Affiliations:** 1 Institute of Brain Science, National Yang-Ming University, Taipei, Taiwan; 2 Brain Research Center, National Yang-Ming University, Taipei, Taiwan; 3 Infection and Immunity Research Center, National Yang-Ming University, Taipei, Taiwan; 4 Genomics Research Center, Academia Sinica, Taipei, Taiwan; 5 Institute of Clinical Medicine, School of Medicine, National Yang-Ming University, Taipei, Taiwan; 6 Institute of Microbiology and Immunology, National Yang-Ming University, Taipei, Taiwan; 7 Department of Medical Research and Education, Taipei Veterans General Hospital, Taipei, Taiwan

**Keywords:** Alzheimer's Disease, Neuroinflammation, Decoy Receptor 3, M2a microglia

## Abstract

**Background:**

Microglia mediate amyloid-beta peptide (Aβ)-induced neuroinflammation, which is one of the key events in the pathogenesis of Alzheimer’s disease (AD). Decoy receptor 3 (DcR3)/TNFRSF6B is a pleiotropic immunomodulator that promotes macrophage differentiation toward the M2 anti-inflammatory phenotype. Based on its role as an immunosupressor, we examined whether DcR3 could alleviate neuroinflammation and AD-like deficits in the central nervous system.

**Method:**

We crossed human APP transgenic mice (line J20) with human DcR3 transgenic mice to generate wild-type, APP, DcR3, and APP/DcR3 mice for pathological analysis. The Morris water maze, fear conditioning test, open-field, and elevated-plus maze were used to access their cognitive behavioral changes. Furthermore, the pathological and immune profiles were examined by immunostaining, ELISA, Q-PCR, and IP. In vitro assays were designed to examine DcR3-mediated innate cytokine profile alteration and the potential protective mechanism.

**Results:**

We reported that DcR3 ameliorates hippocampus-dependent memory deficits and reduces amyloid plaque deposition in APP transgenic mouse. The protective mechanism of DcR3 mediates through interacting with heparan sulfate proteoglycans and activating IL-4^+^YM1^+^ M2a-like microglia that reduces Aβ-induced proinflammatory cytokines and promotes phagocytosis ability of microglia.

**Conclusion:**

The neuroprotective effect of DcR3 is mediated via modulating microglia activation into anti-inflammatory M2a phenotype, and upregulating DcR3 expression in the brain may be a potential therapeutic approach for AD.

**Electronic supplementary material:**

The online version of this article (doi:10.1186/s13024-017-0173-0) contains supplementary material, which is available to authorized users.

## Background

Alzheimer’s disease (AD) is the most common incurable neurodegenerative disease. One of the pathological hallmarks of AD is the extracellular amyloid plaques composed of amyloid-beta peptide (Aβ) which is generated by proteolytic cleavage of amyloid precursor protein (APP). The abnormal accumulation of Aβ is considered to be a critical factor in AD pathogenesis [1, 2]. The aggregation of Aβ into small oligomers and fibrillar plaques triggers neuroinflammation that contributes to the neuronal loss and cognitive decline [1, 3]. Although suppression of chronic inflammation has been proposed as a new direction for AD intervention, the therapeutic effects of anti-inflammatory drugs in current clinical trials are far from satisfactory [4]. Therefore, a novel strategy is necessary to protect neurons from Aβ-induced neurotoxicity and neuroinflammation to preserve memory.

Microglia serve as the first line of host defense in the brain. Microglia activation can be beneficial or detrimental in AD pathogenesis via removing Aβ by phagocytosis or producing pro-inflammatory cytokines that damage neurons [5, 6]. The activated microglia are classified into M1 inflammatory (classical) and M2 anti-inflammatory (alternative) phenotypes [7]. The M1 phenotype can be triggered by lipopolysaccharides, interferon-γ, and Aβ. They produce pro-inflammatory cytokines, such as IL-1β and TNF-α [8, 9], to kill pathogens and induce cytotoxicity [10]. In contrast, the M2 microglia reduce Aβ plaque deposition and alleviate memory impairments in an AD mouse model [11]. Therefore, modulation of microglia activation and differentiation is a potential approach to regulate neuroinflammation in AD [11, 12]. M2 phenotype microglia can be divided into M2a-d subtypes according to the surface and intracellular markers [13, 14]. The M2a subtype microglia (YM1^+^, FIZZ1^+^, CCL17^+^, arginase-1^+^) are induced by IL-4, IL-13, fungal and helminth infections and are capable of suppressing inflammation [15]. However, the functions of these microglia subtypes are still controversial assayed in different in vitro systems [16–18].

Decoy receptor 3 (DcR3)/TNFRSF6B is a soluble decoy receptor which can neutralize the biological functions of three members of tumor necrosis factor superfamily: Fas ligand (FasL) LIGHT, and TL1A to reduce cell death [19–21]. In addition to its neutralizing effect, DcR3 interacts with heparan sulfate proteoglycans (HSPGs) to promote the differentiation of M2-like macrophages through epigenetic regulation [22–24]. DcR3 upregulates the expression of M2 macrophage markers (mannose receptor/CD206, arginase-I, YM-1, CD86, MMP7 and MMP-9) and downregulates the expression of M1 markers (iNOS, CD80, FcγR, IL-6, and TNF-α) [24, 25]. DcR3 transgenic mice are resistant to type-I diabetes and Th17-mediated autoimmune diseases [23]. However, the role of DcR3 in Aβ-mediated neuroinflammation in the brain has not yet been identified.

Given that DcR3 exerts anti-apoptotic and immune-modulatory effects via neutralizing FasL and non-deoy functions we asked whether DcR3 ameliorates AD-like functional deficits and pathological changes using both in vivo and in vitro systems. Here, we demonstrated that Aβ-induced cognitive deficits and neurodegeneration were improved by DcR3 in transgenic mice overexpressing a mutated human APP minigene (hAPP/J20 line). DcR3 skewed microglia differentiation to IL-4^+^YM1^+^ M2a-like subtype, modulated neuroinflammation, conserved synaptic density, and reduced Aβ. Our observations suggest that DcR3 may become a promising reagent for the treatment of AD in the future.

## Methods

### Mice

Hemizygous hAPP transgenic mice (line J20) express an alternatively spliced human APP minigene with the Swedish and Indiana familial AD mutations driven by the PDGF promoter [26]. Hemizygous DcR3 transgenic mice express human DcR3 driven from the CD68 promoter in macrophages/microglia/monocytes [25]. Female DcR3 transgene mice were crossed with male APP transgenic mice to obtain wild-type DcR3 single transgenic, APP single transgenic, and APP/DcR3 double transgenic mice. The littermates of these mice were examined in behavioral tests at 6 months of age and sacrificed for pathological examinations at 6 or 12 months of age.

### Morris Water Maze

The water maze consisted of a water pool (122 cm in diameter) containing opaque water and a platform (10 cm in diameter) submerged 1 cm below the water surface. The hidden platform test consisted of 10 sessions over 5 days and each session comprised three 60-s trials with 15-min inter-trial intervals. The platform location remained constant during the hidden platform sessions, and the entry points were changed semi-randomly between days. One day after the final day of hidden platform training section, a probe trial was conducted by removing the platform and allowing mice to explore in the pool for 1 min. The quadrant in which the platform was previously located was defined as the target quadrant, and the proportion of time (as a percentage) that the each mouse spent in the target quadrant was used to measure memory retention. The number of platform crossings and swim speed were recorded and analyzed with the EthoVision video tracking system (Version 3.1 Noldus Wageningen, Netherlands).

### Fear conditioning

During the day 1 training section mice were habituated in a conditioning box (Graphic State 2.101 Contents, Coulbourn Instruments, PA, USA) for 5 min and then received five pairs of an 8-s tone and a 2-s shock (0.4 mA) followed by a 2-min resting interval. On day 2 testing sections, the trained mice were placed back to the same testing box, and their freezing time was scored for 5 min to measure the contextual conditioned fear response. The cued test was conducted 5 min after the contextual test. Mice were habituated for 5 min in a novel-shaped box and then exposed to three 10-s auditory cues followed with a 2-min resting interval. The freezing times of each mouse were scored during all testing sessions.

### Open field

To detect spontaneous locomotor activity mice were placed in an open chamber (24.32 × 24.32 cm^2^). Their horizontal movement was detected by a 16 × 16 infrared photo-beam arrays placed 1.5 cm above the bottom of the chamber for 15 min (Version 2.0, TRU Scan Photobeam LINC, Coulbourn Instruments, PA, USA).

### Elevated plus maze

The elevated plus-shaped maze consisted of two open arms and two closed arms. All mice were individually placed at the center of the maze and allowed to explore for 10 min. The time spent and distances traveled on each arm were calculated with the Etho Vision video tracking system.

### Immunofluorescence and thioflavin-S staining

Paraformaldehyde-fixed brains were sliced coronally by using a microtome (Leica SM2010R Heidelberg, Germany) and were stored in cryoprotectant medium (30% glycerol, 30% ethylene glycol in PBS) at -20 °C. For immunohistochemistry (IHC) staining, brain slices were blocked in a TBS-buffered solution containing 1% glycine, 0.4% Triton X-100, 10% FBS (FBL01, Caisson labs, USA), 0.1% sodium azide (13412, Sigma, MO, USA) and 3% serum bovine albumin for 2 h and then incubated for 24 h at 4 °C with anti-Iba1 (019–19741, Wako), anti-YM1 (01404, Stem Cell technology), anti-synaptophysin (04–1019, Millipore), anti-MAP2 (MAB378, Millipore) and anti-Aβ (SIG-39320, 6E10, Covance) to measure the distribution of microglia, M2a activated microglia, pre-synaptic density, neuronal density and the total level of Aβ. After incubation, the slices were incubated for 2 h with Alexa594-labeled (111–585–003, Jackson ImmunoResearch) and Alexa488-labeled secondary antibodies (115–546–003, Jackson ImmunoResearch) at room temperature.

For thioflavin-S staining brain slices were incubated with 0.015% thioflavin-S (T1892; Sigma MO, USA) for 15 min at room temperature. All chemicals unless otherwise stated were purchased from Bio Basic Inc. (Canada).

For immunocytochemistry staining primary cells were fixed with 4% paraformaldehyde to measure the degeneration of primary neurons and the morphology changes of microglia in responses to different treatment conditions. Fixed cells were stained with anti-MAP2 or anti-Iba1 antibody to visualize the structure changes. The stained slices or cells were imaged with a fluorescence microscope (Axio Observer A1; Zeiss Germany) or a confocal microscope (Fluoview FV10i; Olympus USA). Images were analyzed with MetaMorph® Microscopy Automation & Image Analysis Software (Molecular Devices, CA, USA).

To quantify the total or M2a microglia surrounding plaques slices were double stained with 6E10 and anti-Iba1 or anti-YM1 antibodies. Plaque areas were circled to determine the centers. The circles were then enlarged 10 μm in radius from the center, which was considered to be the periphery area for measuring the microglia or secreted YM1 coverage.

### Enzyme-linked immunosorbent assays (ELISAs)

For DcR3 measurement up to 500 μl of blood was collected from the facial vein at the submandibular area and was centrifuged at 1,000 g for 15 min to isolate the serum. Serum DcR3 concentrations were measured with a human DcR3 Duo Set (DY142, R&D, USA).

For Aβ measurement the hippocampus of each mouse was homogenized in 5 M guanidine/5 mM Tris (pH 8.0) buffer and diluted with 0.25% casein blocking buffer to a final concentration of 0.5 M guanidine with protease inhibitor (04693116001, Roche, Basel, Switzerland). The levels of total Aβ and Aβ42 were quantified using Aβ ELISA kits (27729 and 27711, IBL, Hamburg, Germany).

For cytokines measurement diluted hippocampus lysates and conditioned media were applied to TNF-α, IL-1β, and IL-6 ELISA kits (555268, 559603, 555240, BD System, NJ, USA). For YM1 measurement, the hippocampus was homogenized in diluting reagent provided by ELISA kit at the concentration of 20 μg tissue/μl, and YM1 concentration were measured using mouse YM1/Chitinase 3-like 3 DuoSet ELISA (DY2446, R&D, USA).

### Quantitative real-time PCR (Q-PCR)

The RNA from the hippocampus and the primary microglia were purified using the Total RNA Mini Kit (Geneaid Taiwan) or TRI reagent (T9424, Sigma, MO, USA), and then immediately reverse transcribed into cDNA by MMLV high-performance reverse transcriptase (RT80125K, Epicentre, WI, USA). The mRNA expression levels were analyzed by using primers (listed in Additional file 1: Table S1) mixed with SYBR Green PCR Master Mix (10476600, Roche, Penzberg, Germany). A StepOnePlus Real-Time PCR System (Applied Biosystem, ABI, MA, USA) was used to monitor the changes of fluorescence intensity from PCR products. GAPDH was used as internal control. The data were analyzed using StepOne software version 2.0.

### Immunoprecipitation (IP)

Cortexes from 12-month-old mice were homogenized with a pestle at the concentration of 1 μg tissue /9 μl HEPES buffer (1% CHAPS 50 mM HEPES, 10 mM EDTA, 150 mM NaCl, pH 7.4). Tissue lysates were centrifuged at 600 × g for 5 mins and supernatants were collected. 200 μl samples were pre-cleared with 50 μl protein G bead (LSKMAGG02, Millipore, Germany) rotating at room temperature for 30 mins. Pre-cleared lysates were incubated with anti-DcR3 (33302, Biolegend, CA, USA), anti-syndecan-1 (10593–1-AP, ProteinTech, IL, USA), anti-glypican-1 (sc-66910, Santa Cruz biotechnology, TX, USA), or anti-Aβ (6E10, SIG-39320, COVANCE, NJ, USA) antibodies at 4 °C overnight, and were then mixed with protein G beads rotating at room temperature for 1 h. The beads were washed with 0.1% Tween 20 in PBS for 20 mins and were eluted by SDS-sample buffer (87.5 mM Tris-HCl, 1% SDS, 30% glycerol, 0.6 M DTT, 180 μM bromphenol blue, pH 6.8) at 95 °C, 10 mins. The eluted samples and input controls were monitored by Western blot.

### Gel electrophoresis and Western blotting analysis

Proteins were separated via 10% or 15% Tris-glycine SDS-polyacrylamide gel electrophoresis and transferred to nitrocellulose membranes. The membranes were probed with rabbit anti-PSD95 (3450 Cell Signaling, MA, USA), mouse anti-DcR3 (33302, Biolegend, CA, USA), rabbit anti-syndecan-1 (10593–1-AP, ProteinTech, IL, USA), rabbit anti-glypican-1 (sc-66910, Santa Cruz biotechnology, TX, USA), mouse anti-Aβ (6E10, SIG-39320, COVANCE, NJ, USA), anti-YM1 (01404, Stem Cell technology, Vancouver, Canada), mouse anti-GAPDH (60004-1-Ig, ProteinTech, IL, USA), and mouse anti-actin (MAB1501, Millipore, MA, USA) antibodies. The membranes were washed and probed with the HRP-conjugated goat anti-mouse IgG and goat anti-rabbit IgG (12–349, AP132P, Millipore, MA, USA). Protein signals were developed by using a chemiluminescent substrate ECL detection system (WBKLS0500, Millipore, MA, USA) and quantified by using a luminescence imaging system (LAS-4000, Fujifilm, Japan).

### Oligomeric Aβ (oAβ) fibrillar Aβ (fAβ) and DcR3 preparation

HFIP-treated Aβ1-42 peptides (rPeptide Inc. A–1163–2, GA, USA) were dissolved in 10% DMSO at 100 μM and stored at –80 °C. Before the experiment, the stock was aged at 4 °C for 24 h to generate oAβ or aged at 37 °C for 18 days to generate fAβ. A DcR3-SAS and Vector-SAS stable line were generated by transfecting the human DcR3 gene or control vector into SAS cells. Culture medium was collected after 24 h of DcR3-SAS and Vector-SAS growth for the conditioned medium experiment.

### Primary neuron and microglia preparation and conditioned medium (CM) stimulation

Primary microglia were prepared from postnatal day 0–5 C57B6/J mice. The cortexes were digested with 100 U papain and 400 U DNase I in HBSS buffer at 37 °C for 30 min. Digested cells were passed through a 70-μm cell strainer (Corning NY, USA). Mixed cortical cells were grown in DMEM-F12. After 21 days incubation, microglia were isolated from a 30:37:70% Percoll (P4937, Sigma. MO, USA) gradient and were seeded in 25-T flasks for 24 h.

Primary cortical neuron cultures were prepared from postnatal day 0–1 C57B6/J mice as primary microglia but seeded at a density of 4x10^5^ per well for 7 days in the neurobasal medium.

To obtain conditioned medium (CM) microglial culture were stimulated with the DcR3-SAS medium before, together or after incubating with Aβ for 72 h and their media were collected as pre-, co- or post-treatment Aβ/DcR3-CM. Aβ-CM was collected from microglial culture stimulated with Vector-SAS medium and Aβ for 72 h, and control-CM was collected from microglial culture stimulated with the vector-SAS medium. For the DcR3 immune-depletion control, the DcR3-SAS medium was incubated with anti-DcR3 (33302, Biolegend, CA, USA) and protein G bead (LSKMAGG02, Millipore, Germany) rotating at 4 °C overnight. DcR3 depleted SAS medium was collected for microglia treatment. For the competition assay, microglia were co-treated with DcR3 and 30 μg/ml heparin sulfate (HS) to interfere the DcR3-HSPG interaction for 8 h and then incubated with Aβ for 72 h. These conditioned media were collected and applied to primary neurons for 72 h.

### Cell survival

Neuronal survival rate after different CM treatment was assessed using MTT (3006 Biotium Inc., CA, USA) and propidium iodide (PI) staining assays according to the manufacturer’s instructions. For the MTT assay, formazan was solubilized in lysis buffer (10% SDS and 20 mM HCl), and the concentration was determined according to the optical density at 570 nm with a Sunrise™ absorbance reader with Magellan™ data analysis software (Version 6; Tecan Switzerland).

For PI staining neurons were incubated with 10 μg/ml PI in PBS for 20 min and were fixed in 4% paraformaldehyde for immunofluorescence staining with the MAP2 antibody. The staining results were quantified as the ratio of PI^+^ neurons to total MAP2^+^ neurons by using MetaMorph® Microscopy Automation & Image Analysis Software (Molecular Devices, CA, USA).

### Mouse cytokine array

The cytokines in the primary microglia conditioned medium were detected by using the mouse cytokine array C1000 (AAM-CYT-1000 RayBiotech, GA, USA). Membranes were incubated with control CM, Aβ-CM or Aβ/DcR3-CM (pre-treatment condition) for 16 h and detected with a Biotin-Streptavidin system. Signals were scanned by using a Fujinon LAS-4000 system and quantified by using Multi Gauge V3.0 software (Fujifilm Corporation, Tokyo, Japan). The level of each cytokine in the control group was set as 100.

### Microglial phagocytosis assay

Purified microglia were seeded at a density of 1x10^5^ cells/well on poly-d-lysine coated coverslips. Attached microglia were treated with oAβ or oAβ + DcR3 for 72 h. Their phagocytic ability was examined by incubating with red fluorescent carboxylated microspheres (F8821 1 μm in diameter, Polysciences Life Technologies, USA) coated with fetal calf serum at 37 °C for 30 min. After three PBS washes, microglia were fixed with 4% paraformaldehyde and stained with anti-Iba1 antibody to visualize the number of engulfed microspheres in the microglia.

### Statistical analysis

Data are presented as the mean ± s.e.m. from at least three independent experiments and were analyzed using Prism software (GraphPad). Differences between data sets were analyzed by unpaired two-tailed Student's *t*-tests or one-way ANOVA followed by the Bonferroni post hoc test. During multiple contrast analysis, the alpha was set as 0.05 (95% confidence intervals). All the precise numbers of samples and their statistical analysis methods of each experiment are listed in Additional file 2: Table S2. A *p* value less than 0.05 was considered to be statistically significant.

## Results

### DcR3 protects against Aβ-induced cognitive deficits and synaptic loss

To investigate the effects of DcR3 on the functional and pathological features of AD transgenic mice overexpressing mutated human APP (line J20) and human DcR3 were crossed to generate APP/DcR3 double transgenic mice. The levels of the full-length APP (FL-APP) in APP and DcR3/APP mice did not change, and nor did the level of DcR3 in DcR3 and DcR3/APP mice (Additional file 3: Figure S1). Because this line of APP mice develops memory impairments and Aβ plaques at the age of 4 months and 5 months, respectively [26, 27], we followed the behavioral changes and Aβ plaque formation in APP/DcR3, APP, DcR3, and wild-type (WT) littermates, respectively, to determine whether DcR3 modulates the pathogenesis of AD at 6 months after birth.

**Fig. 1:**
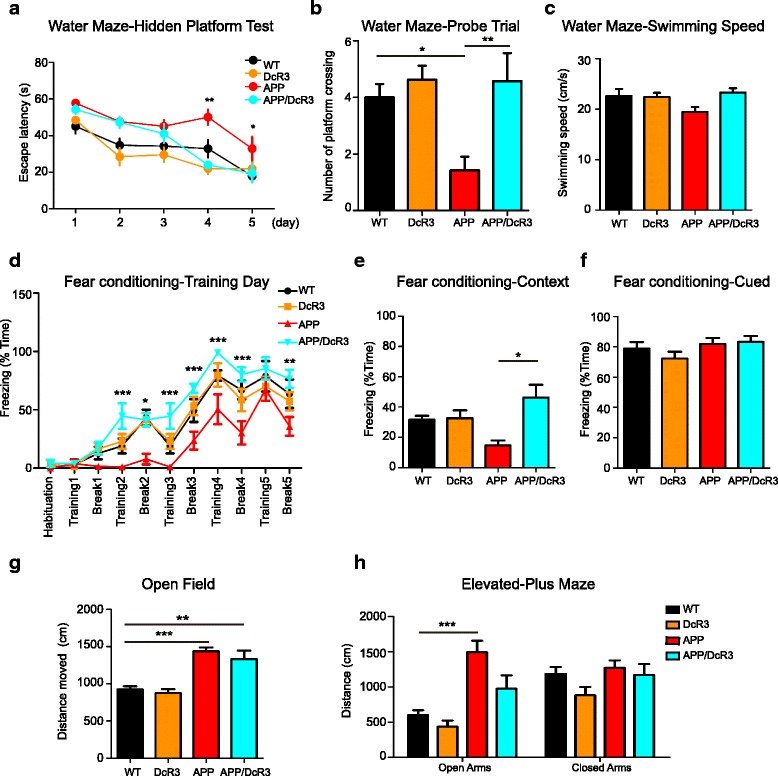
DcR3 improved the hippocampus-related cognitive deficits in APP mice. Mice were subjected to **a**-**c** the Morris water maze test and **d**-**f** the fear conditioning test. **a** The mean daily escape latencies calculated from six trials per day in the hidden platform test. **b** The number of times that the mice crossed the original platform location in the probe trial. **c** Swimming speed during probe trials. **d** Freezing behavior during training day of fear conditioning test. **e**, **f** Percent of time freezing in the **e** contextual test and **f** cued test on the second day. **g** Distance moved in the open field test during 15 min. **h** Distance traveled in the open or closed arms of the elevated plus test during 10 min. *N =* 5-11 mice per genotype. *P ≤ 0.05, **P ≤ 0.01, ***P ≤ 0.001

The Morris water maze was used to assess the spatial learning and memory deficits among these four genotypes of mice (Fig. 1a & b). During the 5 days of the hidden platform test the APP transgenic mice spent more time than WT mice to locate the platform, indicating their deficits in memory acquisition. In contrast, the APP/DcR3 double transgenic mice use less time than the APP transgenic mice to reach the platform at last two days (Fig. 1a). At the 6th day of the probe trial, deficits in memory retention were observed in the APP transgenic mice but not in the APP/DcR3 double transgenic mice (Fig. 1b) compared with WT mice. No significant difference in swimming speeds was found among the 4 genotypes of mice (Fig. 1c). This observation suggested that overexpression of DcR3 rescued spatial learning and memory deficits in 6-month-old APP transgenic mice.

Contextual fear conditioning and auditory-cued fear conditioning tests were further applied to evaluate hippocampus-dependent and amygdala-dependent emotional memory respectively. During the training day, impaired learning was observed in the APP transgenic mice but not in the APP/DcR3 double transgenic mice (Fig. 1d). On day 2 of testing, the APP/DcR3 mice displayed a longer freezing time than the APP mice in the contextual fear conditioning test (Fig. 1e), suggesting that DcR3 reversed the hippocampus-dependent fear memory deficits. In contrast, there was no difference in the amygdala-dependent cued fear conditioning test among the 4 genotypes of mice (Fig. 1f). This observation suggests that DcR3 could ameliorate Aβ-induced hippocampus-related memory deficits.

We further examined the spontaneous motor activity and anxiety levels of these mice in the open field test and in the elevated plus maze. Consistent with previous findings [27] the APP mice traveled a longer distance in the open field and spent more time in the open arm of the elevated plus maze. In these two tests, the APP/DcR3 mice also had higher locomotor activity and lower anxiety-like behavior similar to the APP mice (Fig. 1g and h). These observations suggest that DcR3 reverses hippocampus-dependent memory impairment without changing locomotion- and anxiety-related behaviors.

**Fig. 2:**
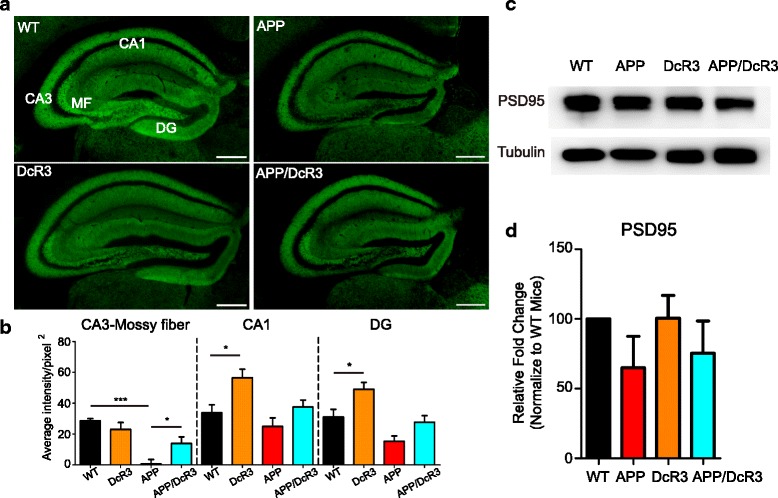
DcR3 restored the pre-synapse marker expression in APP mice. **a** Representative immunofluorescence synaptophysin images in the hippocampus. MF: mossy fiber; DG: dentate gyrus. *Scale bar: 100 μm.* **b** Quantification of synaptophysin intensity in CA3-mossy fibers, CA1, and the DG regions. *N =* 7-14 slices from at least 3 mice per genotype. **c** Representative Western blot imaging of PSD95 among four genotypes of mice and **d** averaged intensity of PSD95. *N =* 5 mice per genotype. *P ≤ 0.05, **P ≤ 0.01, ***P ≤ 0.001

The decline in the cognitive functions in AD patients or mouse models is accompanied by a loss of synaptic markers such as synaptophysin and PSD95 [26, 28, 29]. We thus investigated whether DcR3 prevented the loss of synapses by examining the synaptophysin and PSD95 density (Fig. 2a-d). Compared with WT littermates, significant loss of synaptophysin in the mossy fiber-CA3 pathway was observed in the APP mice as revealed by immunohistochemistry (IHC) staining (Fig. 2a, b & Additional file 4: Figure S2a), while synaptophysin expression level was reversed in the APP/DcR3 mice (Fig. 2a, b & Additional file 4: Figure S2a). Moreover, the synaptophysin intensity in the CA1 area and the dentate gyrus of DcR3 mice was higher than that of WT littermates, suggesting DcR3 is able to upregulate synaptophysin expression even without the presence of Aβ plaques. However, the levels of post-synaptic marker PSD95 and MAP2^+^ neuron had no significant difference among all 4 genotypes of mice (Fig. 2c & d and Additional file 4: Figure S2b & S2c). Because the mossy fiber pathway is critical for memory formation [30], the reversal of synaptophysin loss in this area suggests that DcR3 could preserve synapses to improve spatial memory in the APP mice [31].

### DcR3 mediates neuronal protection via microglia

**Fig. 3:**
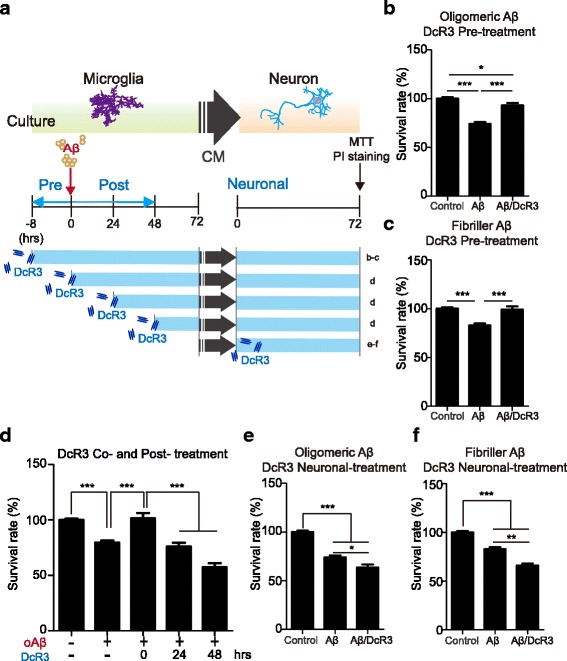
DcR3 suppressed Aβ-induced neurotoxicity in primary neuronal cultures. **a** Schematic of in vitro Aβ and DcR3 treatment conditions. Microglia were stimulated with **b**, **d**, **e** oligomeric and **c**, **f** fibrillar Aβ for 72 h with the addition of DcR3 at different time points, and their conditioned media (CM) were collected for treating onto primary neurons. The survival rates of primary neurons after 72 h incubating with different CM were determined by MTT assay. **b**-**c** The survival rate of primary neuron treated with CM from microglia exposing to DcR3 at 8 h before stimulating with Aβ. **d** The survival rate of primary neuron treated with CM from microglia exposing to DcR3 at 0, 24 and 48 h after stimulating with Aβ. **e**, **f** The survival rate of primary neuron treated with Aβ-CM in addition with DcR3. N ≥ 3 independent experiments. *P ≤ 0.05, **P ≤ 0.01, ***P ≤ 0.001

Because microglia are the major players in Aβ-induced neurotoxicity we asked whether microglia are involved in DcR3-mediated neuronal protection. To address this question, primary microglia were pre-incubated with recombinant DcR3 or control medium for 8 h, followed by exposing to oligomeric or fibrillar Aβ (oAβ or fAβ) for 72 h (DcR3 pre-treatment condition). These Aβ/DcR3-stimulated conditioned media (CM) were harvested and incubated with primary neuronal cells for 72 h (Fig. 3a). The survival rate of the neuronal cells was determined by MTT assay (Fig. 3b & c) and PI staining (Additional file 5: Figure S3a & S3b). Compare with the Aβ-CM, the survival rate was significantly increased in the Aβ/DcR3-CM-treated neurons. To further observe neurotic dystrophy in these CM-treated neurons, the anti-MAP2 antibody was used to detect the morphology of neurons. More dystrophic neurites and swelling structure (arrow) were observed in the Aβ-CM than Aβ/DcR3-CM treated neurons (Additional file 5: Figure S3c), suggesting DcR3 modulated-CM prevented the loss of synaptic process in response to Aβ-induced stress.

We further examined the protective effect of DcR3 by incubating neurons with DcR3-CM simultaneously or after exposure to Aβ respectively (Fig. 3a, DcR3 co- and post-treatment). Compared with the Aβ-CM, the survival rate was significantly increased in neurons co-treated, but not post-treated, with DcR3-CM (Fig. 3d). These results suggested that DcR3-CM-mediated protection is via preventing but not reversing Aβ-induced neurotoxic effect (Fig. 3b-d & Additional file 5: Figure S3a & S3b). To further distinguish whether the neuroprotective effect of DcR3 was contributed from microglia or neuron, primary neurons were incubated with Aβ-CM and recombinant DcR3 for 72 h (Fig. 3a, neuronal treatment). Under this condition, DcR3 failed to protect primary neuron against Aβ-induced neurotoxicity (Fig. 3e & f). These observations suggest that the DcR3-mediated neuronal protection is via modulating microglia activation, rather than promoting neuronal resistance to Aβ-CM.

To examine the quality of oAβ and fAβ structures western blot was applied to examine the aggregation states of Aβ before, after microglial treatment, and after neuronal treatment. The oligomeric (10-72 kDa) and fibrillar (in the stacking gel) structures remain the major species in oAβ and fAβ group respectively (Additional file 6: Figure S4a-c). To further confirm the importance of DcR3 in neuroprotection, DcR3 in the SAS medium were removed by the anti-DcR3 conjugated protein G beads before treating onto microglia (Additional file 6: Figure S4d). While we treated the neuron with the DcR3-depleted CM, the neuronal survival rate was not returned to normal, which indicated a critical role of DcR3 on modulating innate-related cytokine-induced cytotoxicity (Additional file 6: Figure S4e).

### DcR3 reduces amyloid plaque deposition and enhances of Aβ uptake

**Fig. 4:**
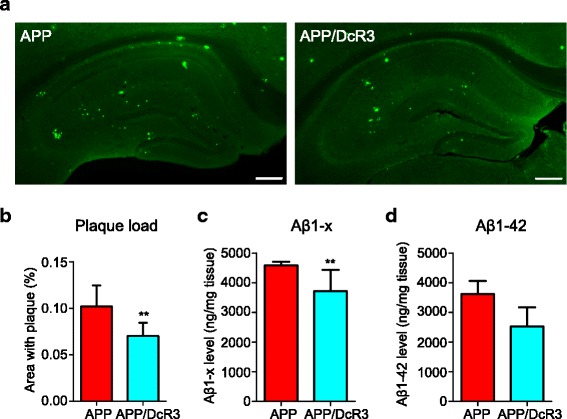
DcR3 reversed amyloid pathology in APP mice. **a** Representative thioflavin-S staining images in coronal brain sections from 12-month-old APP and APP/DcR3 mice. *Scale bar: 100 μm.* **b** Quantification of the percentage of area covered by amyloid plaques in the hippocampi of APP and APP/DcR3 mice. *N =* 24–34 slices from 4–6 mice per genotype. **c**, **d** The hippocampal levels of guanidine soluble **c** total Aβ1-x and **d** Aβ1-42 in APP and APP/DcR3 mice were measured via ELISA. *N =* 6 mice per genotype. **P ≤ 0.01 versus APP mice

Next, we investigated whether DcR3 improves pathological changes by measuring the amyloid plaque deposition and Aβ levels in mice. Compared with APP mice, less β-sheet amyloid plaques were observed in the hippocampus of APP/DcR3 mice as determined by thioflavin-S staining (Fig. 4a & b). In contrast, total Aβ deposition had no significant difference between APP and APP/DcR3 mice as determined by the 6E10 antibody (Additional file 7: Figure S5a-c). Furthermore, the guanidine-soluble total Aβ and Aβ1–42, which play major synaptotoxic roles in AD [26], were decreased in the hippocampus of APP/DcR3 mice as determined by ELISA (Fig. 4c & d). All the evidence indicated that DcR3 reduces amyloid with β-sheet structure and guanidine-soluble Aβ in APP mice.

**Fig. 5:**
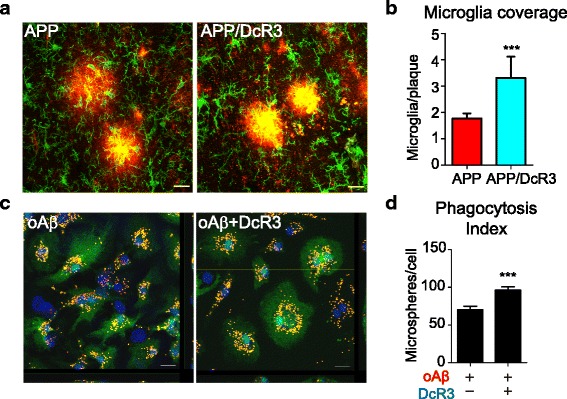
DcR3 modulated the phagocytic ability of microglia. **a** Representative confocal IHC images of Iba1+ microglia (*green*) and Aβ plaques (*red*) in APP and APP/DcR3 mice. *Scale bar: 20 μm.* **b** Quantification of colocalization of activated microglia and plaques. The ratio is calculated as (area of microglia)/(area of plaque) in the region of interest. Number of plaques analyzed: APP = 342, APP/DcR3 = 324 from at least 3 mice per genotype. ***P < 0.001 versus APP. **c** Representative confocal images of oAβ- or oAβ + DcR3-treated primary microglia with engulfed microspheres. *Scale bar: 10 μm.* **d** The number of microspheres engulfed per cell was quantified by using MetaMorph. *N =* 74–76 cells per condition. ***P ≤ 0.001 versus oAβ group

We further examined the Aβ phagocytic activity of microglia which are first recruited to amyloid plaques before being activated to engulf Aβ to clear amyloid plaques in vivo [32]. We observed that clusters of Iba1-positive microglia (green) were found adjacent to the amyloid plaques (red) in the hippocampi of the APP mice. Interestingly, DcR3 enhanced microglia recruitment (Fig. 5a). Reconstruction analysis showed a higher percentage of microglia and amyloid plaques co-localization in APP/DcR3 than that in the APP mice (Fig. 5b). This observation supports the argument that DcR3 enhances the recruitment of activated microglia to clear amyloid plaques.

We further asked whether DcR3 directly promotes the Aβ-induced phagocytic ability of primary microglia by in vitro culture system. To determine phagocytic ability oAβ- or oAβ + DcR3-treated primary microglia were incubated with fluorescence labeled microspheres. In the presence of DcR3, the phagocytic activities of the microglia were upregulated significantly (Fig. 5c & d). These observations suggest that DcR3 enhances the phagocytosis of microglia, which may enhance Aβ-clearance ability.

### DcR3 enhances the IL-4^+^YM1^+^ M2a-like microglia population and anti-inflammatory signaling through binding with HSPGs

Although no change in microglia survival rate was found there was an obvious difference in the morphology of primary microglia at 72 h after Aβ or Aβ/DcR3 treatment in vitro. The morphology of microglia without any treatment is in fusiform and ramified shape, which are characteristics of resting microglia. After oAβ treatment for 72 h, microglia enlarged and became an amoeboid shape. In oAβ/DcR3 treated microglia, the size of amoeboid shape microglia is smaller than the oAβ group (Additional file 8: Figure S6). These observations demonstrated the potent modulatory effect of DcR3 to attenuate oAβ-induced microglia activation.

**Fig. 6:**
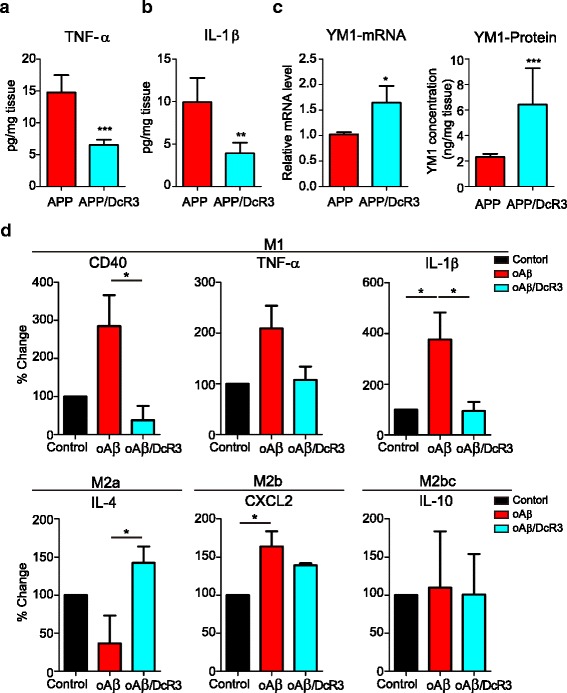
DcR3 enhanced the IL-4^+^YM1^+^ M2a-like subtype of microglia activation in vivo and in vitro. **a**-**c** The APP/DcR3 mice had lower **a** TNF-α and **b** IL-1β levels but higher **c** YM1 mRNA and protein level than APP mice in the hippocampus. *N =* 6–16 mice per genotype. ***P ≤ 0.001; **P ≤ 0.01; *P ≤ 0.05 versus APP mice. **d** Cytokine array determined the cytokine levels in the conditioned medium. Levels of each cytokine in the control CM was arbitrarily set as 100%. *N =* 3 per group. *P ≤ 0.05

Because the change of microglial population can be detrimental or beneficial in neuroinflammation we further investigated the phenotype of microglia activated by Aβ and DcR3 in vivo and in vitro. The expression of cytokines was determined by ELISA, while the markers of type I and type II microglia and components of inflammasome-related proteins were measured by qPCR (Fig. 6a-c and Additional file 9: Figure S7). Compared with APP mice, the expression of TNF-α and IL-1β, which are secreted by activated M1 microglia, were downregulated in APP/DcR3 mice (Fig. 6a & b). In contrast, the expression of YM1 and CCL17, the surface markers for M2a microglia [9], was upregulated by DcR3 (Fig. 6c & Additional file 9: Figure S7a & Additional file 10: Figure S8a-b). Nevertheless, the expression of other inflammatory markers, including the innate immune markers of M2b (IL-6, IL-10), M2c (IL-10, TGF-β, arganise1, CD206) [9, 16], and inflammasome (NLRP3, ASC, IL-18) [11], were similar (Additional file 9: Figure S7b-i). Furthermore, YM1 intensity near the plaques was higher and became more condensed in the APP/DcR3 mice than that in APP mice (Additional file 10: Figure S8a & S8b), indicating that microglia recruited to the plaques are polarized toward M2a-like subtype.

Since the sources of TNF-α and IL-1β in the brain are not only from microglia we confirmed the role of DcR3 to modulate the secretion of cytokines from microglia by using in vitro culture system. The cytokine profiles in the Aβ-CM or Aβ/DcR3-CM (from Fig. 3a, pre-treatment condition) were analyzed by cytokine array (Fig. 6d). Compared with Aβ-CM, lower levels of pro-inflammatory mediators (CD40, TNF-α, IL-1β, IL-12) with higher the levels of the M2a inducer (IL-4) and the M2a marker (YM1) were observed in Aβ/DcR3-CM (Fig. 6d and Additional file 11: Figure S9a). In contrast, the levels of M2b and M2c markers (CXCL2 and IL-10) were not altered by DcR3 (Fig. 6d). All these observations suggest that DcR3 has the potent effect to modulate cytokine secretion by modulating the activation and differentiation of microglia. It is interesting to note that DcR3 did not alter the expression of MMP9, which contributes to plaque clearance in the brain [33], suggesting MMP9-dependent proteolytic degradation of Aβ was not influenced by DcR3 (Additional file 11: Figure S9b). The complete list of all the changes from this cytokines array is listed in Additional file 12: Table S3. Thus, we concluded that DcR3 is able to skew microglia differentiation into IL-4^+^YM1^+^ M2a-like microglia in vivo and in vitro.

**Fig. 7:**
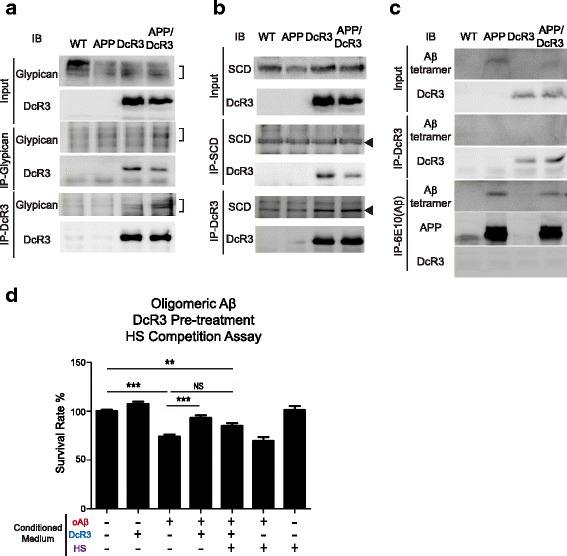
DcR3 interacted with HSPG to protect neuron under Aβ stress. **a**-**c** Co-immunoprecipitation to determine the interaction between DcR3 with **a** glypican, **b** syndecan (SCD), and **c** Aβ or APP. **d** Heparan sulfate (HS) competition treatment to block the protective function of DcR3 against Aβ stress in vitro. *N =* 3 per group. ***P ≤ 0.001; **P ≤ 0.01; NS, not significant

Because the interaction between DcR3 and HSPG is critical for modulating macrophage activation in vitro [24] we asked whether DcR3 also interacts with glypicans and syndecans, which are the most abundant HSPGs to modulate myeloid cell differentiation in the brain [22, 34, 35]. We found that DcR3 interacts with glypican-1 (Fig. 7a) and syndecan (Fig. 7b) by co-immunoprecipitation assay. In contrast, DcR3 did not interact with Aβ or APP (Fig. 7c), thus excluding the possibility that the neuroprotective effect of DcR3 is via direct neutralization of Aβ or APP. The interaction between DcR3 and HSPGs suggested that human DcR3 may modulate the activation and differentiation of microglia via interacting with HSPGs in vivo. To further confirm the role of HSPG in DcR3-mediated protection against Aβ-induced neurotoxicity, heparin sulfate (HS) was used to block DcR3-HSPG interaction by a competition assay as described in Fig. 4a [22]. In the presence of HS, DcR3-mediated neuroprotective effect against Aβ was attenuated (Fig 7d), suggesting DcR3-HSPG interaction contributes partially against Aβ-induced toxicity [36].

## Discussion

**Fig. 8:**
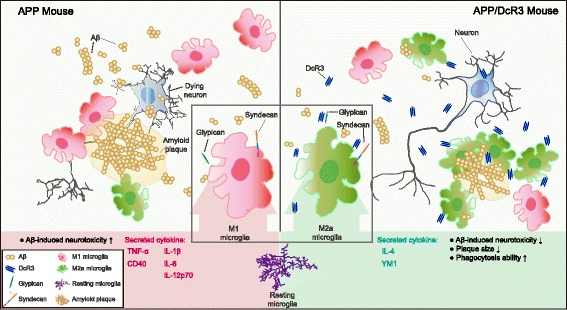
Working model: In the presence of Aβ, microglia polarize toward the M1 phenotype and secrete pro-inflammatory cytokines, which trigger neurodegeneration. DcR3 interacts with HSPGs and drives microglia polarization to the IL-4^+^YM1^+^ M2a-like subtype that secrete more anti-inflammatory cytokines. This change enhances Aβ phagocytosis, thereby reducing amyloid plaques and cognitive deficits in APP mice

In this study a human secreted protein DcR3 prevented Aβ-induced functional and pathological deficits in both in vivo and in vitro AD models. Three potential mechanisms involve in DcR3 neuroprotective effect against amyloid pathogenesis (Fig. 8). First, under Aβ stress, DcR3 induces IL-4^+^YM1^+^ M2a-like microglia that reduce the pro-inflammation cytokines to prevent neurotoxicity. Second, DcR3 enhances microglia recruitment to plaques and phagocytic efficiency to clear Aβ. Finally, DcR3 interacts with surface HSPGs. This interaction may eliminate Aβ-HSPGs downstream cytotoxicity or inhibit the HSPGs-mediated inflammatory responses [37, 38].

### DcR3 promotes anti-inflammatory effect

The importance of microglia-neuron interaction has been implicated in many neuroinflammatory-related disorders [39]. In the pre-plaque AD mouse oAβ and complement C1q initiate complement cascade and recruit microglia via CR3 to eliminate synapses [40]. Manipulation of the innate immune system into alternatively M2 activated microglia has been considered as a promising therapeutic strategy for AD [11, 41]. For example, intracerebral injections of IL-4/IL-13 or IL-33 reverse memory deficits and reduce Aβ plaque load in AD mouse models [41, 42]. In addition, YM1^+^ cells could protect neurons during acute brain injury [43, 44]. We found that DcR3-triggered IL-4^+^YM1^+^ M2a-like microglia contribute to anti-inflammatory response and functional recovery in vitro and in vivo. DcR3 reduces Aβ − induced pro-inflammatory cytokines, TNF-α and IL-1β [45], to prevent severe neuroinflammation and neurodegeneration [5]. Although DcR3 has been shown its’ ability to polarize macrophage differentiation in the periphery [24, 25], this study first presents that DcR3 modulates the complex innate immunity profiles and is able to counter Aβ-induced neuroinflammation in the brain.

In disease-related models microglia activation has dynamic, multidimensional and mosaic signatures [46]. Generally speaking, switching activated microglia from M1 into M2 anti-inflammatory spectrum can reverse inflammatory-related diseases [12, 47]. However, microglia/ macrophage have diverse characteristics in different regions and switch dynamically in response to environmental changes [46, 48]. Studies focusing on macrophage/microglia activation in specific tissues or using particular cytokine stimulation in vitro might not reveal the complex profiles of innate immunity environment [49, 50]. In addition, the M1/M2 microglia classification has been questioned on modulating neurotrophic factors to regulate synaptic plasticity and memory [48, 51, 52]. Therefore, more evidence are needed to better understand the roles of microglia populations toward AD pathogenesis.

### DcR3 promotes phagocytosis

To remove plaque via phagocytosis activated microglia are recruited to the site of plaques in AD animal models and patients [32, 53]. This recruitment is important for engulfing amyloids or tissue debris through lysosome-dependent manner or for constituting a barrier to prevents neurotoxic protofibrillar Aβ42 [32, 54, 55]. Although both astrocyte and microglia could be found near the plaques, only microglia have the ability to degrade Aβ [32]. Inefficient phagocytosis ability of microglia has been reported in AD animal models, and down-regulating phagocytosis-related gene expression has been found in AD subjects [56, 57]. This deficit could be caused by Aβ aggregates, reactive oxygen species (ROS), and pro-inflammatory mediators such as TNF-α, IL-1β, IL-6, and IL-18 [58, 59]. Regulating microglia phagocytosis is considered as a detrimental way to prevent tissue damage in the brain [60]. We found that DcR3 induces more microglia activation near plaque regions (Fig. 5a-b) and enhances microglia phagocytosis to remove Aβ in vivo and in vitro (Fig. 4 & Fig. 5c-d). The enhanced phagocytic ability of Aβ + DcR3-stimulated microglia may reverse the deficit of reducing phagocytic cells in AD patients or AD mice models [61, 62]. Altogether, our results suggest a potential beneficial role of DcR3 on modulating microglia into anti-inflammatory phagocytosis status.

### DcR3 interacts with HSPG to regulate neuronal survival

We found that DcR3 interacts with mouse surface HSPGs glypican-1 and syndecan-1 (Fig. 7a & b), and DcR3 neuroprotective effect can be shaded by heparin in vitro (Fig. 7d). The HSPGs on the microglia and oligodendrocytes near lesions could modulate the accumulation of senile plaques and neurofibrillary tangles. [63, 64]. Glypican-1 can bind to Aβ aggregates to upregulate ER stress and to stimulate microglia activation that results in enhanced cytotoxicity [34, 65]. Syndecans are involved in the production of Aβ [66], ROS [35], and inflammatory cytokines [35, 67]. Furthermore, deletion of HSPGs accelerates Aβ clearance in APP/PS1 mice, suggesting that inhibition of Aβ-HSPG interaction is able to suppress Aβ-induced neuroinflammation [64]. However, the addition of heparin did not completely reverse DcR3 protection effects, which may due to the multiple roles of heparin such as promoting amyloidogenesis and modulate neuroinflammation [68, 69]. Because DcR3 interacts with HSPGs but not Aβ in our AD mouse model (Fig. 7a-c), DcR3-mediated neuronal protection may compete Aβ binding to HSPG or suppress HSPGs-CD14/TLR4 mediated inflammation [38]. The functions of HSPGs binding domain in DcR3 and its contributions to innate systems requires further analysis.

### The anti-inflammatory treatments using cytokines and DcR3

Several clinical studies have shown contradictory evidence for non-steroidal anti-inflammatory drugs (NSAIDs) in the treatment or prevention of AD [70]. These NSAIDs cannot be used in high doses or for a long period of time because of potential side effects in the cardiovascular or gastrointestinal systems [71]. In all NSAID clinical trials, cognitive performance had no significant improvements, and inconsistent results were reported for indomethacin [72, 73], ibuprofen [74, 75], celecoxib [76], rofecoxib [77, 78], and naproxen treatments [76]. A potential reason for the failure of anti-inflammatory approaches is that different anti-inflammatory cytokines induce diverse responses. For example, one anti-inflammatory cytokine, IL-10, facilitates Aβ aggregation, inhibits microglial phagocytic ability, and causes cognitive dysfunction in APP mice [79, 80]. In contrast, the other anti-inflammatory cytokine, IL-4, triggers microglia phagocytosis to reduce Aβ deposition [15, 81]. Thus, the IL-10 induced microglia may be harmful but IL-4^+^ microglia may be beneficial to AD-related symptoms. For novel treatment approaches, it is important to identify the microglia subtypes that ameliorates AD pathogenesis.

In comparison to other anti-inflammatory treatments DcR3 is a non-cytokine that induces an anti-inflammatory response, neutralizes FasL-induced cell death, and reduces ROS production [82]. Therefore, DcR3 may be an alternative agent for designing the future treatment. DcR3 concentration in our transgenic mouse serum is 268.6 ± 59.2 pg/ml (Additional file 3: Figure S1), which is slightly higher than healthy human (63.7 ± 21.9 pg/ml) but similar to the asthma patients (266.1 ± 60.6 pg/mL) [83]. Because DcR3 is highly expressed in the endometrium during pregnancy, it appears to be a safe natural immunomodulator to suppress neuronal inflammation in the presence of dangerous endogenous signals [84]. These findings suggested that DcR3 may be a safe therapeutic agent for early AD and other neuroinflammation-related diseases.

## Conclusions

In summary, our findings are in line with the current idea that switching microglia phenotype modulates amyloid pathogenesis. Especially, we first identified that DcR3 induces phagocytosis ability and creates an IL-4^+^YM1^+^ environment to clear Aβ plaque in the brain. DcR3 might also regulate surface HSPGs activity that mediates Aβ-related neurodegeneration and pro-inflammatory signalings (Fig. 8). Taken together, the DcR3-induced specific IL-4^+^YM1^+^ innate response could broaden our views on AD early-intervention and bring a novel thinking on medical development.

## Additional files

Additional file 1: Table S1. List of the real-time PCR primers sets (5′-3′) for the target genes. (PDF 4549 kb)

Additional file 2: Table S2. Statement on sample size and statistical measures. (PDF 1351 kb)

Additional file 3: Figure S1. Full-length APP and DcR3 expression in four genotypes of mice at 6 months of age. (a, b) Levels of full-length APP did not change between the APP and APP/DcR3 mice (c) Levels of DcR3 did not change between the DcR3 and APP/DcR3 mice (*N =* 18-22 mice per genotype). *P ≤ 0.05. NS, not significant. (PDF 8354 kb)

Additional file 4: Figure S2. Synaptophysin and MAP2 immunostaining in the hippocampus. (a)Enlarged view of synaptophysin staining in Fig. 2a. *Scale bar: 100 μm.* (b)Representative immunofluorescence images labeled with neuronal marker MAP2 in the mouse brain slice. *Scale bar: 100 μm.* (c) Quantification graph comparing the average intensity in CA3-mossy fibers, CA1, and DG region (*N =* 7-13 mice per genotype). (PDF 4774 kb)

Additional file 5: Figure S3. DcR3 protected neurons against Aβ stress in vitro. (a) Representative illustrations of PI staining used to measure the number of dead neurons after CM treatment. Red: dead cells (PI); Blue: nucleus (DAPI). *Scale bar: 20 μm.* (b) The ratio of PI/DAPI indicates the change in the number of dead neurons after treatment with the conditioned medium. (c) Primary neurons were labeled with neuronal markers (MAP2, Green), and nucleus (DAPI, Blue). Arrow indicated broken and swelling neurites. *Scale bar: 20 μm* (PDF 1016 kb)

Additional file 6: Figure S4. Aβ aggregation status and DcR3 immuno-depletion control for the conditioned media experiment. (a-c) Western blotting were applied to determine the aggregation state of Aβ peptide in (a) fresh prepared oAβ or fAβ, (b) after 72 h incubating with microglia, (c) after 72 h incubating with neuron. (d) Representative Western blotting image of DcR3 protein levels after immuno-depletion. (e) DcR3 depletion conditioned treatment had no protective function on neuronal survival under Aβ stress (*n =* 4). *P ≤ 0.05,***P ≤ 0.001. The neuronal survival rate of DcR3 Depletion conditioned media treatment was reduced compared to oAβ/DcR3 treatment. (PDF 524 kb)

Additional file 7: Figure S5. Effect of DcR3 on Aβ deposition in the hippocampus. (a) Immunostaining of total Aβ (6E10, Red) and nucleus (DAPI, Blue) of the hippocampus. *Scale bar: 200 μm.* (b, c) Quantification data of (b) total numbers of Aβ plaques and (c) plaque coverage (*N =* 4 mice per genotype, *N =* 8 brain slices per mouse). (PDF 119 kb)

Additional file 8: Figure S6. Morphological changes of primary microglia in vitro under Aβ or Aβ/DcR3 treatment. The representative fluorescent images were labeled with microglia marker (Iba1, red) and nucleus (DAPI, blue) in microglia culture. *Scale bar: 20 μm.* (PDF 8273 kb)

Additional file 9: Figure S7. Expression inflammatory-related genes in mice of four genotypes. The mRNA levels of (a) M2a, (b, c) M2b, (c-f) M2c, and (g-i) inflammasome related proteins were examined by using qPCR. *P ≤ 0.05. (PDF 52 kb)

Additional file 10: Figure S8. DcR3 induced more M2a microglia surrounded plaques and more YM1 expression in vivo. (a) The representative confocal images were labeled with M2a activated microglia marker (YM1, green) and Aβ (6E10, red) in the mouse brain slices. *Scale bar: 20 μm.* (*N =* 13-14 slices per genotype) (b) Quantification of the YM1 positive signal intensity surrounded plaques. ﻿﻿The quantification method for Fig. 5b and Additional file 10: ﻿Figure S8 is showen in Additional file 13: Figure S10.﻿﻿ ***P ≤ 0.001 vs. APP mice. (PDF 72 kb)

Additional file 11: Figure S9. Identifying protein expression patterns in primary microglia lysates and CM. (a) DcR3 promoted more YM1 secretion in the Aβ treated primary microglia culture according to the immunoblotting analysis. GAPDH was used as a loading control. (b) The changes in pro-MMP9 levels in the cytokine array analysis. (PDF 554 kb)

Additional file 12: Table S3. List of the C3 cytokine array data. (PDF 24088 kb)

Additional file 13: Figure S10. Illustration of the quantification method of microglia or YM1 around each plaque in Fig. 5b and Additional file 10: Figure S8. Plaque areas were circled to determine the centers. The circles were then enlarged 10 μm in radius from the center, which was considered to be the region of interest for measuring the microglia or secreted YM1 coverage. (PDF 11009 kb)

## Abbreviations

AD: Alzheimer’s disease
ANOVA: Analysis of variance
APP: Amyloid precursor protein
Aβ: Amyloid-beta peptide
CM: Conditioned medium
DcR3: Decoy receptor 3
ELISAs: Enzyme-linked immunosorbent assays
fAβ: Fibrillar Aβ
FL-APP: Full-length amyloid precursor protein
HS: Heparan sulfate
HSPGs: Heparan sulfate WT, Wild-type
IHC: Immunohistochemistry
IL: Interleukin
NSAIDs: Non-steroidal anti-inflammatory drugs
oAβ: Oligomeric Aβ
PAGE: Polyacrylamide gel electrophoresis
Q-PCR: Quantitative real-time Polymerase Chain Reaction
ROS: Reactive oxygen species
TNFR: Tumor necrosis factor receptor
TNF-α: Tumor necrosis factor alpha
