# Supplementary material for: Amelioration of amyloid-β-induced deficits by DcR3 in an Alzheimer’s disease model
Source: Mol Neurodegener. 2017 Apr 24;12:30. doi: 10.1186/s13024-017-0173-0 (PMC5402663; doi:10.1186/s13024-017-0173-0)
Supplement: Supplementary file 1 — List of the real-time PCR primers sets (5′-3′) for the target genes. (PDF 4549 kb) [file 13024_2017_173_MOESM1_ESM.pdf]

ADDITIONAL FILE 7: FIGURE S7

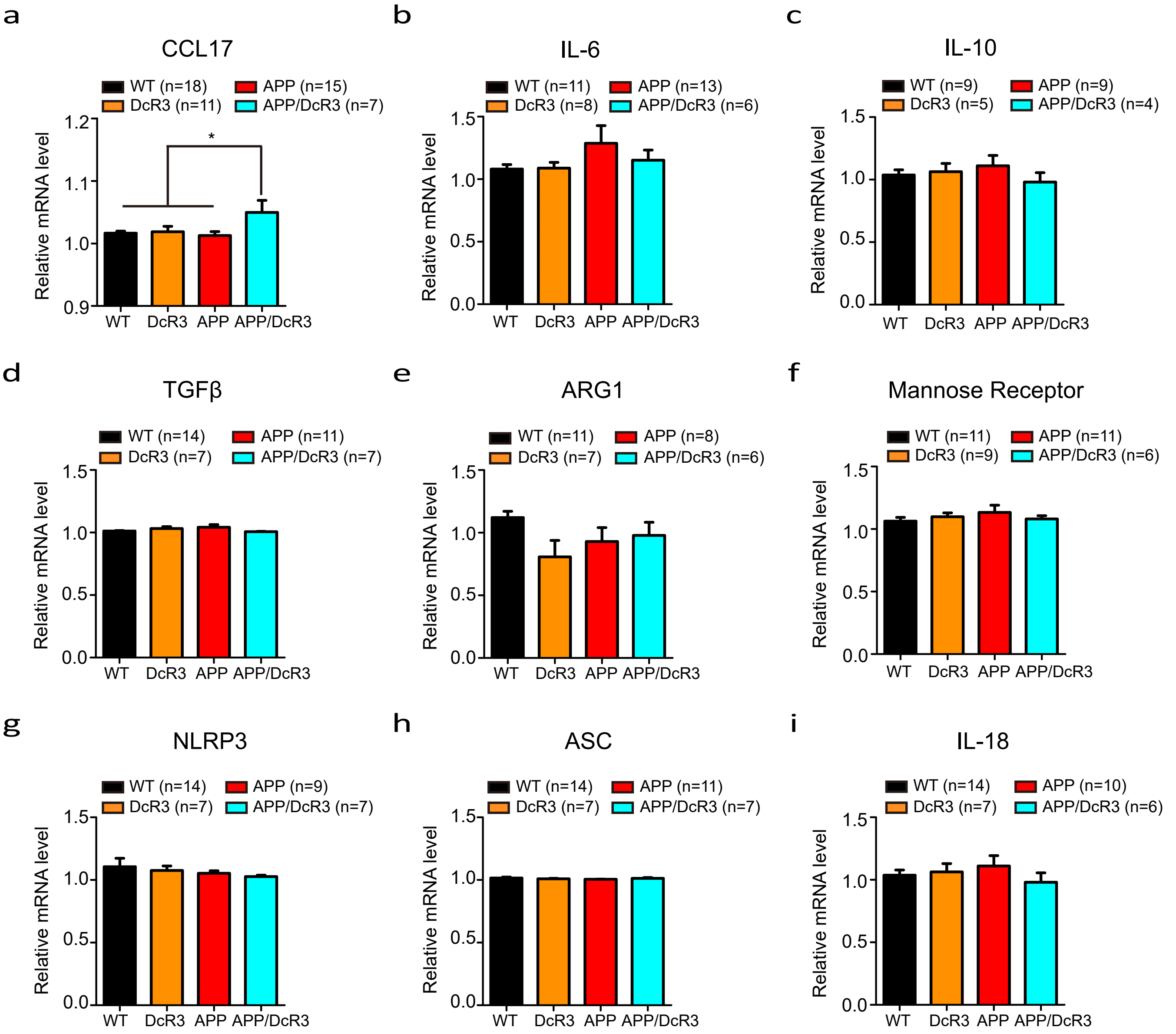

Additional file 7: Figure S7: Expression inflammatory-related genes in mice of four genotypes. The mRNA levels of (a) M2a, (b, c) M2b, (c-f) M2c, and (g-i) inflammasome related proteins were examined by using qPCR. $*P \leq 0.05$.
