# Supplementary material for: Amelioration of amyloid-β-induced deficits by DcR3 in an Alzheimer’s disease model
Source: Mol Neurodegener. 2017 Apr 24;12:30. doi: 10.1186/s13024-017-0173-0 (PMC5402663; doi:10.1186/s13024-017-0173-0)
Supplement: Supplementary file 2 — Statement on sample size and statistical measures. (PDF 1351 kb) [file 13024_2017_173_MOESM2_ESM.pdf]

ADDITIONAL FILE 9: FIGURE S9

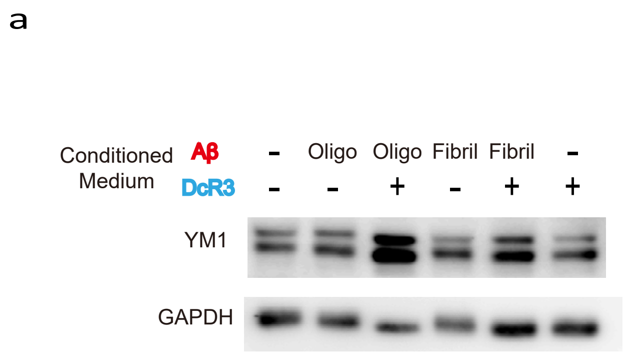

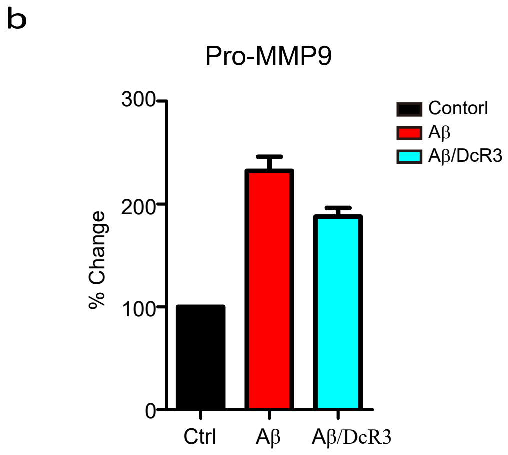

Additional file 9: Figure S9: Identifying protein expression patterns in primary microglia lysates and CM.

(a) DcR3 promoted more YM1 secretion in the Aβ treated primary microglia culture according to the immunoblotting analysis. GAPDH was used as a loading control. (b) The changes in pro-MMP9 levels in the cytokine array analysis.
