# Supplementary material for: Amelioration of amyloid-β-induced deficits by DcR3 in an Alzheimer’s disease model
Source: Mol Neurodegener. 2017 Apr 24;12:30. doi: 10.1186/s13024-017-0173-0 (PMC5402663; doi:10.1186/s13024-017-0173-0)
Supplement: Supplementary file 3 — Full-length APP and DcR3 expression in four genotypes of mice at 6 months of age. (a, b) Levels of full-length APP did not change between the APP and APP/DcR3 mice (c) Levels of DcR3 did not change between the DcR3 and APP/DcR3 mice (N = 18-22 mice per genotype). *P ≤ 0.05. NS, not significant. (PDF 8354 kb) [file 13024_2017_173_MOESM3_ESM.pdf]

Additional file 13: Table S3: List of the C3 cytokine array data.

	MEAN ± SEM		
Target	Control	oAβ	oAβ/DcR3
Axl	100±16.3	0±34.4	153.2±7.9
BLC	100±3.1	192.7±20.4	100.6±3.2
CD30L	100±5.5	268.3±43.6	83.±7.9
CD30	100±7.4	144.7±26.1	128.7±10.5
CD40	100±7.7	169.3±25.7	0±58.7
CRG-2	100±4.1	224.8±31.3	123.9±10.5
CTACK	100±1.4	183.5±15	113.2±5
CXCL16	100±1.1	136.3±6.9	201.3±31.3
Eotaxin1	100±3.9	101.3±14.5	26.1±12.2
Eotaxin2	100±1.4	85.4±4.6	96.7±1.1
FasL	100±2.9	105.2±3.2	95.2±1.8
CX3CL1	100±7.8	139.5±5.8	93.2±2.8
GCSF	100±1.4	197.4±6.7	194.6±4.6
GM-CSF	100±10.3	158.9±13.8	142.5±7.1
IFN-gamma	100±34	0±105.3	0±73.8
IGFBP3	100±2.1	172.1±13.5	136.1±4.5
IGFBP5	100±1.4	113.±5.6	87.6±1.6
IGFBP6	100±1.5	32.5±15.7	70.9±4.3
IL-1alpha	100±2.3	236.4±19.7	112.5±7.5
IL-1beta	100±2.2	232.2±34.3	96.2±5.6
IL-2	100±2.9	278.5±42.9	147.2±13.3
IL-3	100±12.5	457.7±88.2	164.2±21.4
IL-3 Rb	100±4.1	482.8±87.6	260.7±41.1
IL-4	100±1	66.5±12.7	125.5±3.6
IL-5	100±2.7	125.9±11.1	191.7±11
IL-6	100±0.9	214.9±10.2	245.4±12.1
IL-9	100±1.6	211.8±19.4	120.7±3.7
IL-10	100±13.3	211.5±13.1	222.4±10.4
IL-12 p40/70	100±2	4883.6±640.2	4505.9±446.1
IL-12 p70	100±5.5	0±103.2	0±54.9
IL-13	100±1.6	143.±6.1	109.7±4.8
IL-17	100±2.9	136.9±9.4	134.3±20.6
CXCL1	100±0.6	169.5±11	138.1±3.2
Leptin R	100±4.2	883.8±182	445.9±79.1
Leptin	100±0.6	169.9±13.9	171.1±15.7
CXCL5	100±0.7	163.1±14.9	113.5±4.1
L Selectin	100±458.4	23259.8±5345.8	12678.7±3023.1
Ltn/XCL1	100±1	91.4±7.4	104.±2.7
MCP1	100±0.8	110.5±3.4	119.9±2.7

MCP5	100±0.9	119.3±1.8	140.6±3.2
M-CSF	100±2.7	152.3±8.6	127.±8
MIG	100±0.8	122.8±2.9	107.4±6.6
MIP-1 alpha	100±0.6	125.1±3	122.±1
MIP-1 gamma	100±0.9	129.2±3.6	112.8±1.1
MIP-2	100±0.5	166.1±7.2	137.2±1.9
MIP-3 beta	100±0.8	136.2±4.9	92.5±2.4
MIP-3 alpha	100±0.7	209.1±9	174.3±3
PF-4	100±0.9	190.6±8.4	140.5±2.2
P Selectin	100±1.4	209.5±20.6	144.2±11.1
RANTES	100±0.9	118.6±8.1	82.2±3.1
SCF	100±5.3	247.1±32	78.8±7
SDF-1 alpha	100±2.9	110.7±7.4	99.3±5.4
TARC	100±4.3	103.6±6.9	76.5±6.5
TCA-3	100±1.8	113.1±3.7	86.6±4
TECK	100±4.6	92.5±7.6	53.±7.6
TIMP-1	100±2.2	113.6±3.7	98.8±2.1
TNF alpha	100±6.9	109.7±8.5	82.7±11.9
sTNFRI	100±1.8	109.5±3.3	96.2±2.4
sTNFRII	100±2	130.3±5.2	123.4±3.3
TPO	100±3.3	111.1±6.3	81.5±3.6
VCAM-1	100±1	143.4±4.3	109.2±3.2
VEGF	100±2.6	123.2±11.2	70.4±5.8

The average intensity of each target in the control group were set as 100. N≥3 for each group
