# Supplementary material for: Amelioration of amyloid-β-induced deficits by DcR3 in an Alzheimer’s disease model
Source: Mol Neurodegener. 2017 Apr 24;12:30. doi: 10.1186/s13024-017-0173-0 (PMC5402663; doi:10.1186/s13024-017-0173-0)
Supplement: Supplementary file 4 — Synaptophysin and MAP2 immunostaining in the hippocampus. (a)Enlarged view of synaptophysin staining in Fig. 2a. Scale bar: 100 μm. (b)Representative immunofluorescence images labeled with neuronal marker MAP2 in the mouse brain slice. Scale bar: 100 μm. (c) Quantification graph comparing the average intensity in CA3-mossy fibers, CA1, and DG region (N = 7-13 mice per genotype). (PDF 4774 kb) [file 13024_2017_173_MOESM4_ESM.pdf]

ADDITIONAL FILE 10: FIGURE S10

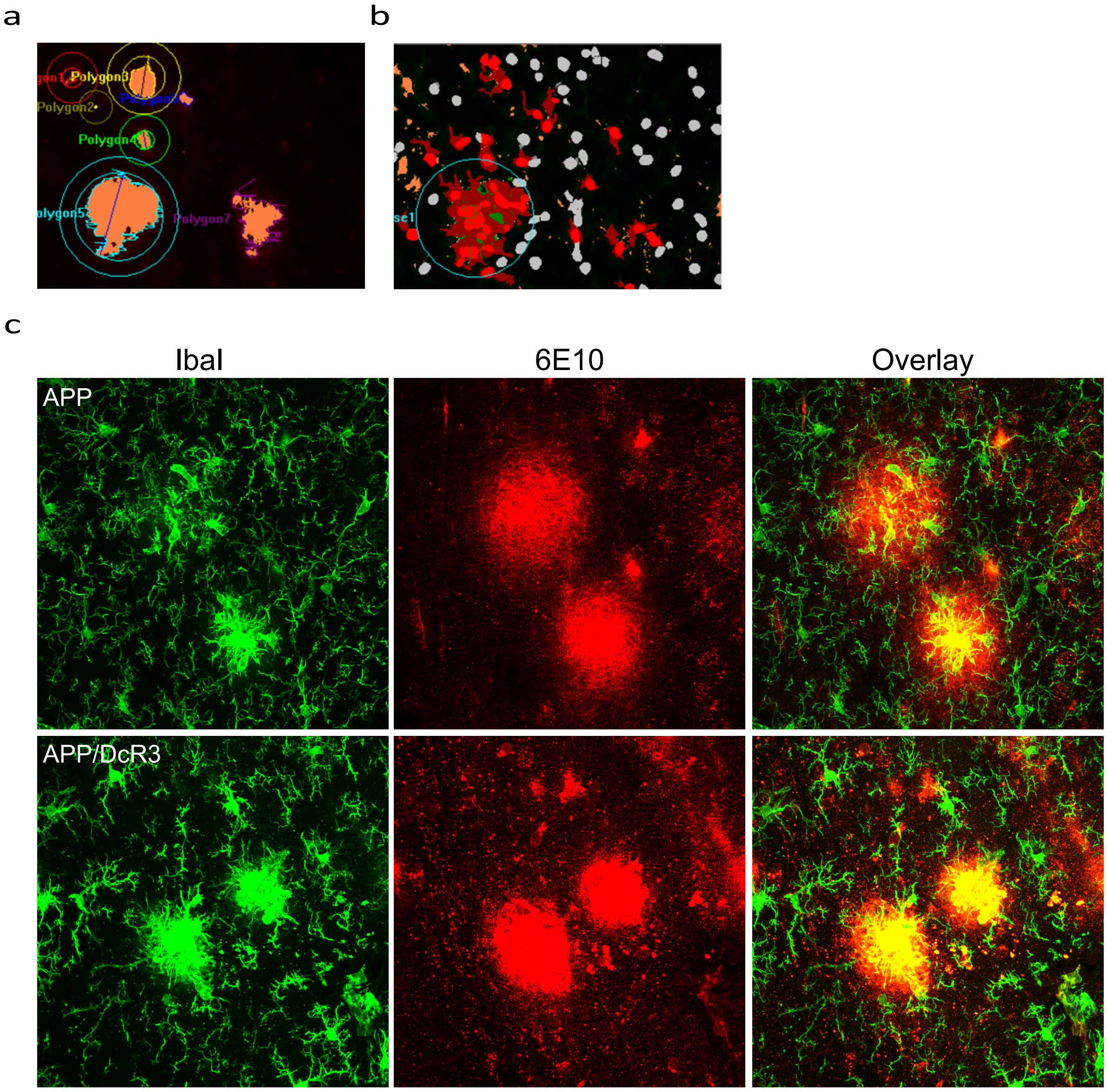

Additional file 10: Figure S10: Illustration of the quantification method of microglia or YM1 around each plaque in Fig. 5b and Additional file 8: Table S8.

Plaque areas were circled to determine the centers. The circles were then enlarged 10 μm in radius from the center, which was considered to be the region of interest for measuring the microglia or secreted YM1 coverage.
