# Supplementary material for: Amelioration of amyloid-β-induced deficits by DcR3 in an Alzheimer’s disease model
Source: Mol Neurodegener. 2017 Apr 24;12:30. doi: 10.1186/s13024-017-0173-0 (PMC5402663; doi:10.1186/s13024-017-0173-0)
Supplement: Supplementary file 5 — DcR3 protected neurons against Aβ stress in vitro. (a) Representative illustrations of PI staining used to measure the number of dead neurons after CM treatment. Red: dead cells (PI); Blue: nucleus (DAPI). Scale bar: 20 μm. (b) The ratio of PI/DAPI indicates the change in the number of dead neurons after treatment with the conditioned medium. (c) Primary neurons were labeled with neuronal markers (MAP2, Green), and nucleus (DAPI, Blue). Arrow indicated broken and swelling neurites. Scale bar: 20 μm (PDF 1016 kb) [file 13024_2017_173_MOESM5_ESM.pdf]

Additional file 11: Table S1: List of the real-time PCR primers sets (5'-3') for the target genes.

Additional file 11: Table S1: Real-Time PCR primer sets(5'-3')

	Forward Primer	Reverse Primer
TNF-α	TCCAGGCGGTGCCTATGT	CGATCACCCCGAAGTTCAGT
IL-1β	GATGATAACCTGCTGGTGTGTGA	GTTGTTCATCTCGGAGCCTGTAG
IL-6	ACCACGGCCTTCCCTACTTC	TCTGTTGGGAGTGGTATCCTCTGT
MR	CCCAAGGGCTCTTCTAAAGCA	AACGCCGGCACCTATCAC
CCL17	CTGCTCGAGCCACCAATGTA	GGAATGGCCCCTTTGAAGTAA
YM1	TTCTGGTGAAGGAAATGCGTAAA	GCAGCCTTGGAATGTCTTTCTC
IL-10	GCCCAGAAATCAAGGAGCATT	CGCATCCTGAGGGTCTTCA
TGFβ	AACCCCCATTGCTGTCCCGTG	GCGCTGAATCGAAAGCCCTGT
ARG1	AGCCGCTGGAACCCAGAGAGA	TGGACCTCTGCCACCACACCA
IL-18	ACTGTACAACCGCAGTAATAC	AGTGAACATTACAGATTTATCCC
ASC	GACAGTACCAGGCAGTTCGT	AGTCCTTGCAGGTCAGGTTC
NLRP3	GTGAAACAAAACGTGCCTTAGAAG	CCACGCCTACCAGGAAATCTC
GAPDH	GCATCCACTGGTGCTGCC	TCATCATACTTGGCAGGTTTC
