# Supplementary material for: Amelioration of amyloid-β-induced deficits by DcR3 in an Alzheimer’s disease model
Source: Mol Neurodegener. 2017 Apr 24;12:30. doi: 10.1186/s13024-017-0173-0 (PMC5402663; doi:10.1186/s13024-017-0173-0)
Supplement: Supplementary file 6 — Aβ aggregation status and DcR3 immuno-depletion control for the conditioned media experiment. (a-c) Western blotting were applied to determine the aggregation state of Aβ peptide in (a) fresh prepared oAβ or fAβ, (b) after 72 h incubating with microglia, (c) after 72 h incubating with neuron. (d) Representative Western blotting image of DcR3 protein levels after immuno-depletion. (e) DcR3 depletion conditioned treatment had no protective function on neuronal survival under Aβ stress (n = 4). *P ≤ 0.05,***P ≤ 0.001. The neuronal survival rate of DcR3 Depletion conditioned media treatment was reduced compared to oAβ/DcR3 treatment. (PDF 524 kb) [file 13024_2017_173_MOESM6_ESM.pdf]

Additional file 12: Table S2: Statement on sample size and statistical measures.

Fig	Sample size	Definitions of statistical methods and measures
**1a, b, c**	n = 9, WT; n = 8, DcR3; n = 7, APP; n = 7, APP/DcR3.	one-way ANOVA
**1d, e, f**	n = 5, WT; n = 11, DcR3; n = 7, APP; n = 10, APP/DcR3.	one-way ANOVA
**1 g, h**	Mouse numbers: n = 40, WT; n = 8, DcR3; n = 37, APP; n = 6, APP/DcR3.	one-way ANOVA
**2a, b**	Slices for CA3: n = 8, WT; n = 7, DcR3; n = 10, APP; n = 10, APP/DcR3.<br>Slices for CA1: n = 14, WT; n = 10, DcR3; n = 13, APP; n = 14, APP/DcR3.<br>Slices for DG: n = 14, WT; n = 12, DcR3; n = 13, APP; n = 13, APP/DcR3.<br>Each quantitative data from at least 3 mice per genotype.	one-way ANOVA
**2 c, d**	n = 5 mice per genotype.	one-way ANOVA
**3b,c,e,f**	Data were obtained from three independent experiments, and each treatment performed in quadruplicating.	one-way ANOVA
**3d**	Data were obtained from more than four independent experiments, and each treatment performed in quadruplicating.	one-way ANOVA
**4 a, b**	Mouse numbers: n = 6, APP; n = 4, APP/DcR3.<br>Slice numbers: n = 34, APP; n = 24, APP/DcR3.	two-tailed unpaired Student's t test.
**4 c, d**	n = 6 mice per genotype.	two-tailed unpaired Student's t test.
**5 a, b**	Number of plaques analyzed: APP = 342, APP/DcR3 =324. Each quantitative data from at least 3 mice per genotype.	two-tailed unpaired Student's t test.
**5 c, d**	Cell numbers: n=76, oAβ; n=74, oAβ+DcR3.	two-tailed unpaired Student's t test.
**6 a, b, c**	Mouse numbers in TNF-α ELISA: n = 14, APP; n =8, APP/DcR3; Mouse numbers in IL-1β ELISA: n = 16, APP; n =8, APP/DcR3; Mouse numbers in YM1 mRNA level: n = 15, APP; n =7, APP/DcR3; Mouse numbers in YM1 ELISA: n = 9, APP; n =6, APP/DcR3;	two-tailed unpaired Student's t test.
**6 d**	n = 3 per treatment	one-way ANOVA
**7d**	Data were obtained from three independent experiments, and each treatment performed in quadruplicating.	One-way ANOVA
**S1 b**	n = 6 mice per genotype.	One-way ANOVA

**S1 c**	Mouse numbers: n = 18, WT; n = 21, DcR3; n = 21, APP; n = 22, APP/DcR3.	One-way ANOVA
**S2 b, c**	Mouse numbers for CA3: n = 11, WT; n = 8, DcR3; n = 13, APP; n = 12, APP/DcR3; CA1: n = 12, WT; n = 8, DcR3; n = 13, APP; n = 12, APP/DcR3; and DG: n = 11, WT; n = 7, DcR3; n = 11, APP; n = 12, APP/DcR3.	One-way ANOVA
**S3**	Experiments were conducted for over 5 times with 3 regions for each treatment.	One-way ANOVA
**S4**	n = 4 per treatment	one-way ANOVA
**S5**	Slice numbers: n = 8<br>Each quantitative data from 4 mice per genotype.	two-tailed unpaired Student's t test.
**S7**	Sample numbers are presented in figures.	one-way ANOVA
**S8 a, b**	Slice numbers: n = 14, APP; n = 13, APP/DcR3.<br>Each quantitative data from 4 mice per genotype.	two-tailed unpaired Student's t test.
**S9 b**	Data were obtained from two independent experiments, and each treatment performed in duplicating.	one-way ANOVA

Data are presented as the mean ±s.e.m. from at least three independent experiments and were analyzed using Prism software (GraphPad) to determine the statistical significance of the difference. Differences between data sets were analyzed by Unpaired, two-tailed Student's *t*-tests or one-way ANOVA followed with the Bonferroni post hoc test. During multiple contrast analysis, the alpha was set as 0.05 (95% confidence intervals). A *p* value less than 0.05 was considered to be statistically significant.
