# Supplementary material for: Amelioration of amyloid-β-induced deficits by DcR3 in an Alzheimer’s disease model
Source: Mol Neurodegener. 2017 Apr 24;12:30. doi: 10.1186/s13024-017-0173-0 (PMC5402663; doi:10.1186/s13024-017-0173-0)
Supplement: Supplementary file 7 — Effect of DcR3 on Aβ deposition in the hippocampus. (a) Immunostaining of total Aβ (6E10, Red) and nucleus (DAPI, Blue) of the hippocampus. Scale bar: 200 μm. (b, c) Quantification data of (b) total numbers of Aβ plaques and (c) plaque coverage (N = 4 mice per genotype, N = 8 brain slices per mouse). (PDF 119 kb) [file 13024_2017_173_MOESM7_ESM.pdf]

ADDITIONAL FILE 1: FIGURE S1

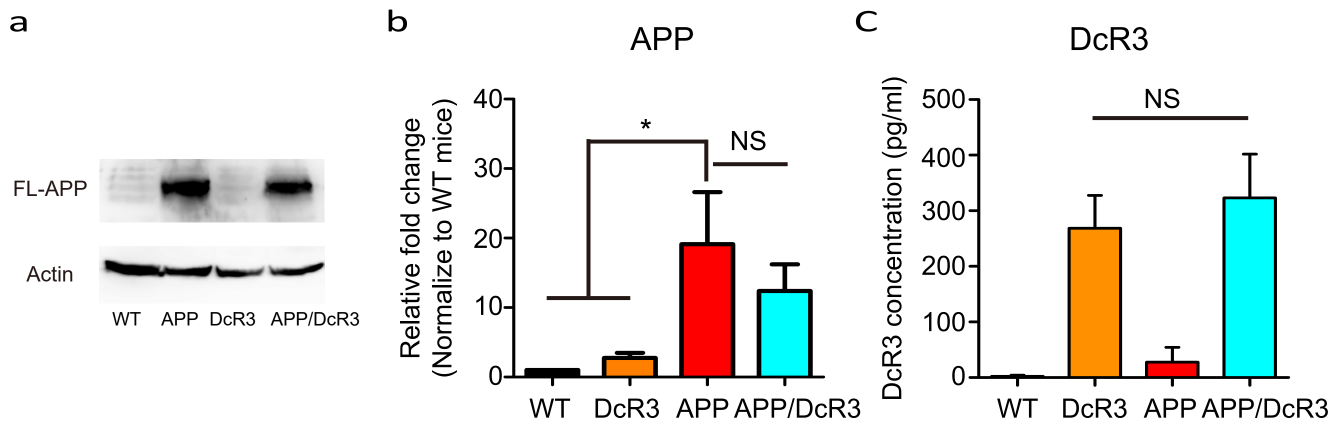

Additional file 1: Figure S1: Full-length APP and DcR3 expression in four genotypes of mice at 6 months of age.

(a, b) Levels of full-length APP did not change between the APP and APP/DcR3 mice (c) Levels of DcR3 did not change between the DcR3 and APP/DcR3 mice (N = 18-22 mice per genotype). $^*P \leq 0.05$. NS, not significant.
