# Supplementary material for: Amelioration of amyloid-β-induced deficits by DcR3 in an Alzheimer’s disease model
Source: Mol Neurodegener. 2017 Apr 24;12:30. doi: 10.1186/s13024-017-0173-0 (PMC5402663; doi:10.1186/s13024-017-0173-0)
Supplement: Supplementary file 8 — Morphological changes of primary microglia in vitro under Aβ or Aβ/DcR3 treatment. The representative fluorescent images were labeled with microglia marker (Iba1, red) and nucleus (DAPI, blue) in microglia culture. Scale bar: 20 μm. (PDF 8273 kb) [file 13024_2017_173_MOESM8_ESM.pdf]

ADDITIONAL FILE 2: FIGURE S2

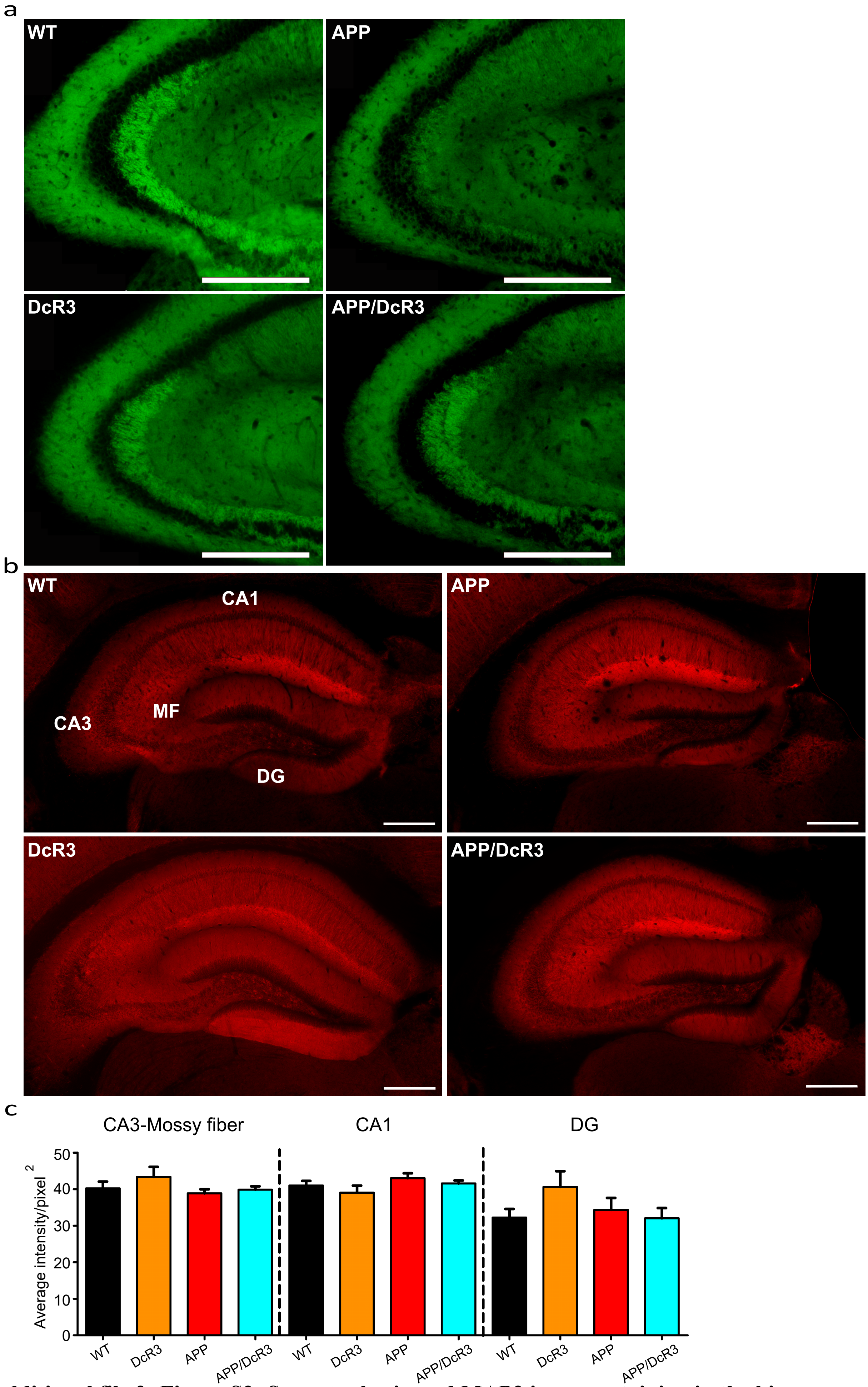

Additional file 2: Figure S2: Synaptophysin and MAP2 immunostaining in the hippocampus. (a)Enlarged view of synaptophysin staining in figure 2a. *Scale bar: 100 µm.* (b)Representative immunofluorescence images labeled with neuronal marker MAP2 in the mouse brain slice. *Scale bar: 100 µm.* (c) Quantification graph comparing the average intensity in CA3-mossy fibers, CA1, and DG region (N = 7-13 mice per genotype).
