# Supplementary material for: Amelioration of amyloid-β-induced deficits by DcR3 in an Alzheimer’s disease model
Source: Mol Neurodegener. 2017 Apr 24;12:30. doi: 10.1186/s13024-017-0173-0 (PMC5402663; doi:10.1186/s13024-017-0173-0)
Supplement: Supplementary file 9 — Expression inflammatory-related genes in mice of four genotypes. The mRNA levels of (a) M2a, (b, c) M2b, (c-f) M2c, and (g-i) inflammasome related proteins were examined by using qPCR. *P ≤ 0.05. (PDF 52 kb) [file 13024_2017_173_MOESM9_ESM.pdf]

ADDITIONAL FILE 3: FIGURE S3

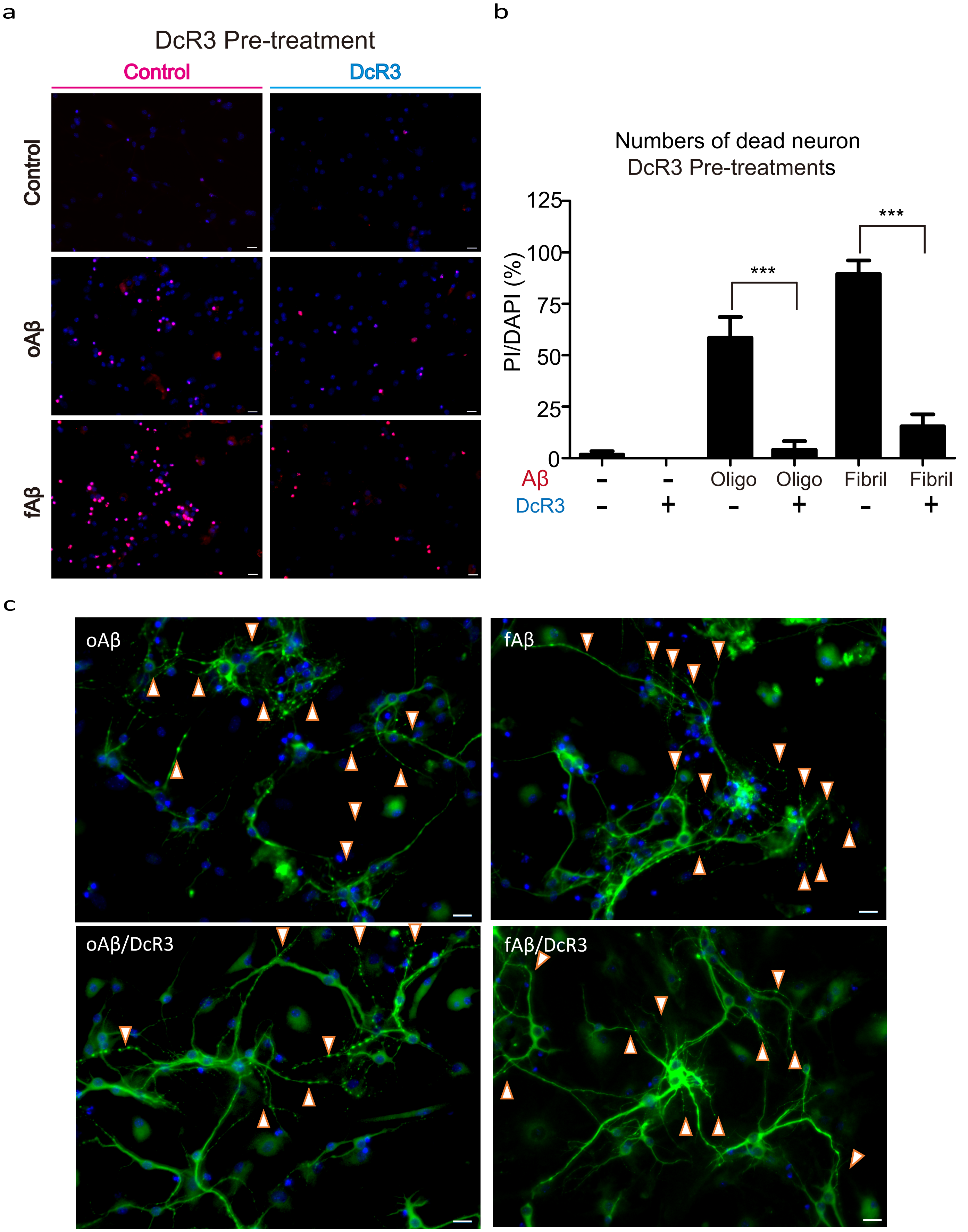

Additional file 3: Figure S3: DcR3 protect neurons against Aβ stress *in vitro*.

(a) Representative illustrations of PI staining used to measure the number of dead neurons after CM treatment. Red: dead cells (PI); Blue: nucleus (DAPI). *Scale bar: 20 μm*. (b) The ratio of PI/DAPI indicates the change in the number of dead neurons after treatment with the conditioned medium. (c) Primary neurons were labeled with neuronal markers (MAP2, Green), and nucleus (DAPI, Blue). Arrow indicated broken and swelling neurites. *Scale bar: 20 μm*.
