# Supplementary material for: Amelioration of amyloid-β-induced deficits by DcR3 in an Alzheimer’s disease model
Source: Mol Neurodegener. 2017 Apr 24;12:30. doi: 10.1186/s13024-017-0173-0 (PMC5402663; doi:10.1186/s13024-017-0173-0)
Supplement: Supplementary file 10 — DcR3 induced more M2a microglia surrounded plaques and more YM1 expression in vivo. (a) The representative confocal images were labeled with M2a activated microglia marker (YM1, green) and Aβ (6E10, red) in the mouse brain slices. Scale bar: 20 μm. (N = 13-14 slices per genotype) (b) Quantification of the YM1 positive signal intensity surrounded plaques. ﻿﻿The quantification method for Fig. 5b and Additional file 10: ﻿Figure S8 is showen in Additional file 13: Figure S10.﻿﻿ ***P ≤ 0.001 vs. APP mice. (PDF 72 kb) [file 13024_2017_173_MOESM10_ESM.pdf]

ADDITIONAL FILE 8: FIGURE S8

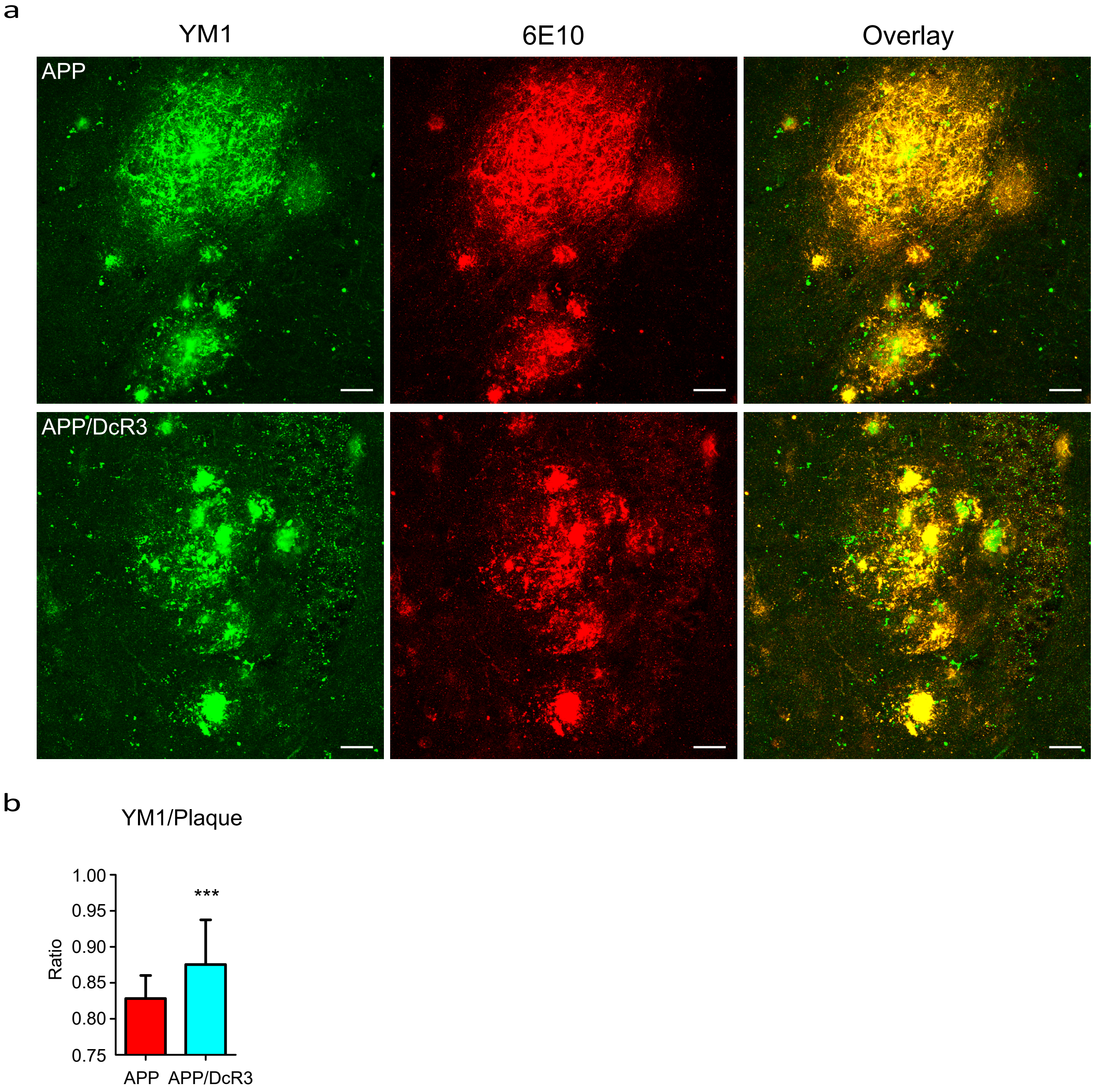

Additional file 8: Figure S8: DcR3 induced more M2a microglia surrounded plaques and more YM1 expression *in vivo*.

(a) The representative confocal images were labeled with M2a activated microglia marker (YM1, green) and Aβ (6E10, red) in the mouse brain slices. *Scale bar: 20 µm.* (N=13-14 slices per genotype) (b) Quantification of the YM1 positive signal intensity surrounded plaques. ***P ≤ 0.001 vs. APP mice.
