# Supplementary material for: Amelioration of amyloid-β-induced deficits by DcR3 in an Alzheimer’s disease model
Source: Mol Neurodegener. 2017 Apr 24;12:30. doi: 10.1186/s13024-017-0173-0 (PMC5402663; doi:10.1186/s13024-017-0173-0)
Supplement: Supplementary file 11 — Identifying protein expression patterns in primary microglia lysates and CM. (a) DcR3 promoted more YM1 secretion in the Aβ treated primary microglia culture according to the immunoblotting analysis. GAPDH was used as a loading control. (b) The changes in pro-MMP9 levels in the cytokine array analysis. (PDF 554 kb) [file 13024_2017_173_MOESM11_ESM.pdf]

ADDITIONAL FILE 4: FIGURE S4

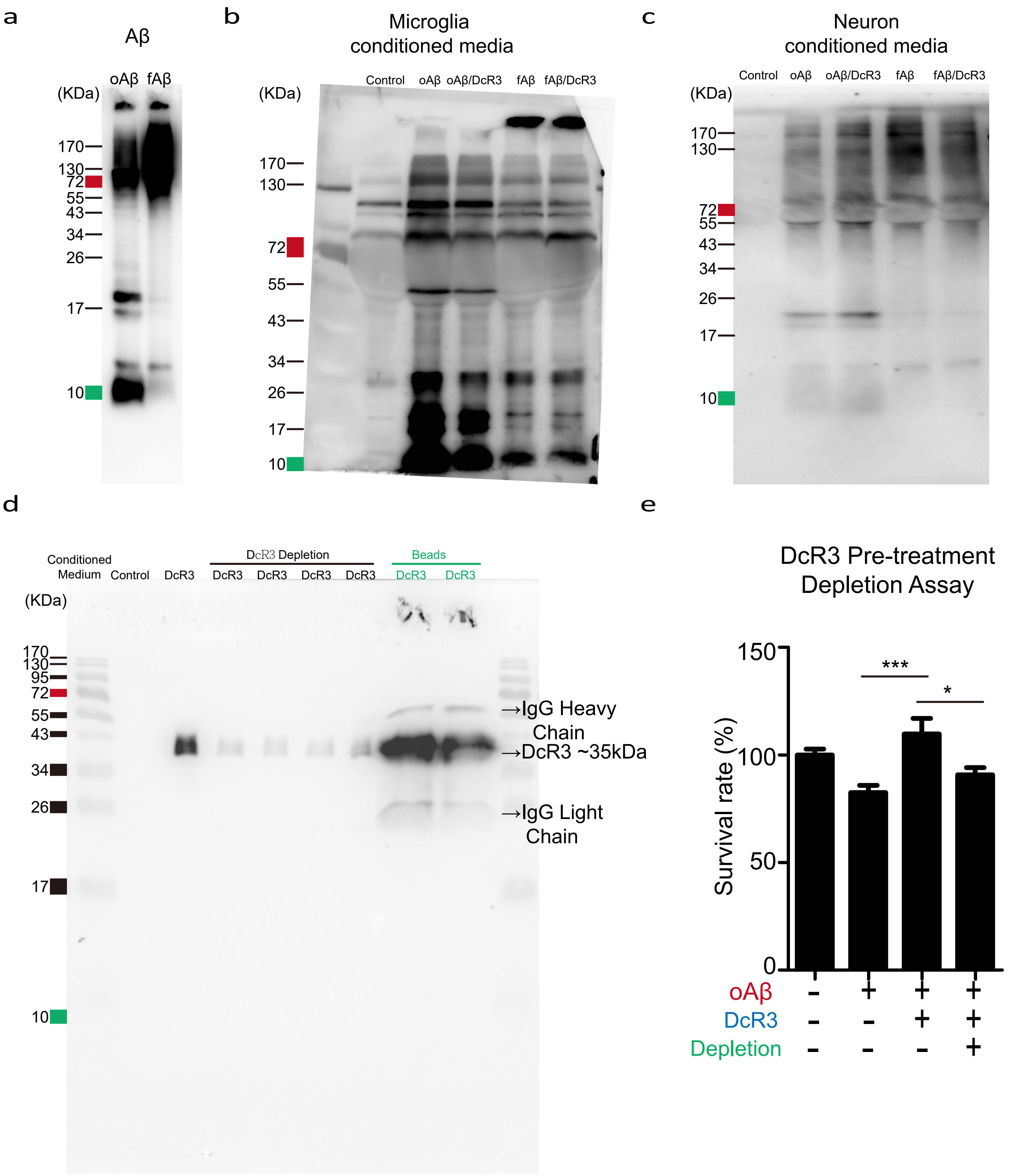

Additional file 4: Figure S4: Aβ aggregation status and DcR3 immuno-depletion control for the conditioned media experiment.

(a-c) Western blotting were applied to determine the aggregation state of Aβ peptide in (a) fresh prepared oAβ or fAβ, (b) after 72 hours incubating with microglia, (c) after 72 hours incubating with neuron. (d) Representative Western blotting image of DcR3 protein levels after immuno-depletion. (e) DcR3 depletion conditioned treatment had no protective function on neuronal survival under Aβ stress (n=4). $^*P \leq 0.05$, $^{***}P \leq 0.001$. The neuronal survival rate of DcR3 Depletion conditioned media treatment was reduced compared to oAβ/DcR3 treatment.
