# Supplementary material for: Amelioration of amyloid-β-induced deficits by DcR3 in an Alzheimer’s disease model
Source: Mol Neurodegener. 2017 Apr 24;12:30. doi: 10.1186/s13024-017-0173-0 (PMC5402663; doi:10.1186/s13024-017-0173-0)
Supplement: Supplementary file 12 — List of the C3 cytokine array data. (PDF 24088 kb) [file 13024_2017_173_MOESM12_ESM.pdf]

ADDITIONAL FILE 5: FIGURE S5

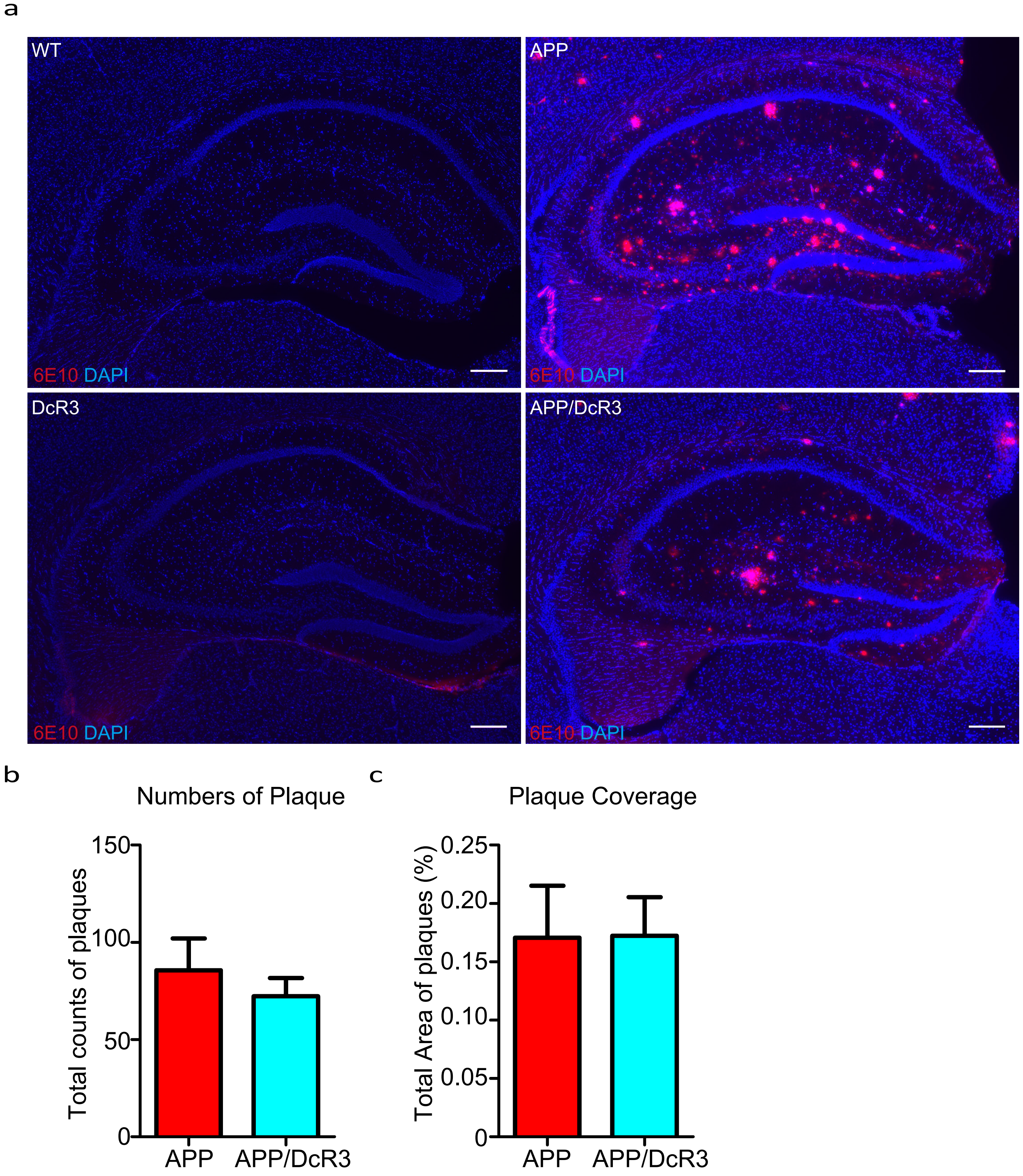

Additional file 5: Figure S5: Effect of DcR3 on Aβ deposition in the hippocampus.
(a) Immunostaining of total Aβ (6E10, Red) and nucleus (DAPI, Blue) of the hippocampus. *Scale bar: 200 μm.* (b, c) Quantification data of (b) total numbers of Aβ plaques and (c) plaque coverage (N = 4 mice per genotype, N=8 brain slices per mouse).
