# Supplementary material for: Amelioration of amyloid-β-induced deficits by DcR3 in an Alzheimer’s disease model
Source: Mol Neurodegener. 2017 Apr 24;12:30. doi: 10.1186/s13024-017-0173-0 (PMC5402663; doi:10.1186/s13024-017-0173-0)
Supplement: Supplementary file 13 — Illustration of the quantification method of microglia or YM1 around each plaque in Fig. 5b and Additional file 10: Figure S8. Plaque areas were circled to determine the centers. The circles were then enlarged 10 μm in radius from the center, which was considered to be the region of interest for measuring the microglia or secreted YM1 coverage. (PDF 11009 kb) [file 13024_2017_173_MOESM13_ESM.pdf]

ADDITIONAL FILE 6: FIGURE S6

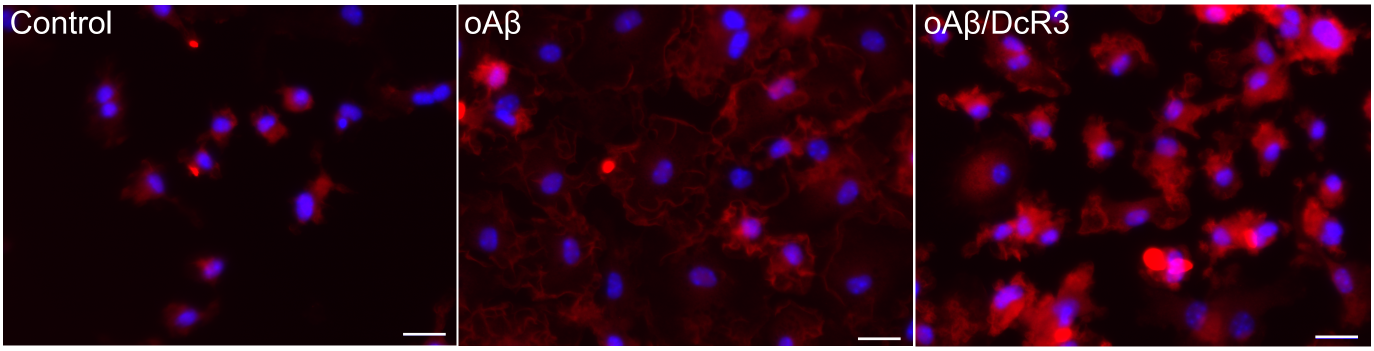

Additional file 6: Figure S6: Morphological changes of microglia *in vitro* under Aβ or Aβ/DcR3 treatment.

The representative fluorescent images were labeled with microglia marker (Iba1, red) and nucleus (DAPI, blue) in microglia culture. *Scale bar: 20 µm*.
